# Stochastic Model Refinement for Multi-GNSS Real-Time PPP Based on Orbit and Clock Extrapolation Error Statistics

**DOI:** 10.3390/s26217061

**Published:** 2026-11-08

**Authors:** Hongchao Li, Jinzhong Mi, Shouzhou Gu

**Affiliations:** 1 School of Geomatics, Liaoning Technical University, Fuxin 123000, China; lihongchao@yrcti.edu.cn; 2 School of Geomatics Engineering, Yellow River Conservancy Technical University, Kaifeng 475004, China; 3 Chinese Academy of Surveying and Mapping, Beijing 100036, China; goldheal@casm.ac.cn; 4 Beijing Fangshan Satellite Laser Ranging National Observation and Research Station, Beijing 102406, China

**Keywords:** real-time precise point positioning, state space representation, data age, short-term outage, orbit and clock extrapolation, direct variance inflation

## Abstract

**Highlights:**

**What are the main findings?**

- Orbit and clock extrapolation errors generally increase with data age and differ among constellations and satellites. The training model and test reference model show broadly similar additional SISRE error scales and age-dependent growth.
- System-level and satellite-level direct variance inflation (DVI) schemes reduce 3D RMS positioning errors by 67.33% and 69.29%, respectively, relative to extrapolation without additional downweighting. Satellite-level DVI provides a further reduction of 5.98% relative to system-level DVI, although this additional benefit is not observed in every station component or outage window.

**What are the implications of the main findings?**

- Historical extrapolation-error statistics provide prior information on additional variances for PPP, supporting offline construction of statistical error models and online dynamic weighting.
- Direct variance inflation based on historical extrapolation-error statistics offers a feasible way to improve PPP accuracy during short-term SSR outages. Satellite-level modeling provides a modest overall benefit, but its additional gain is scenario-dependent.

**Abstract:**

Short-term outages of real-time state space representation (SSR) products degrade precise point positioning (PPP) accuracy. We propose a multi-GNSS PPP stochastic model refinement method based on historical statistics of additional signal-in-space range error (SISRE). System-level and satellite-level age-dependent models are constructed offline from joint range-domain statistics of orbit and clock extrapolation errors. Online, model outputs are converted into equivalent additional variances for direct variance inflation (DVI). Thirty days of Wuhan University SSR data are divided into non-overlapping 15-day training and test sets for modeling and cross-day validation. Within 10 min, BDS-3 and Galileo show relatively small extrapolation errors, GPS shows pronounced inter-satellite differences, and GLONASS shows higher overall errors. The training model and test reference model show similar error scales and age-dependent growth, indicating cross-day repeatability. Without additional downweighting, four-constellation simulated kinematic PPP experiments with 10 min outages yield a three-dimensional root mean square (3D RMS) positioning error of 13.26 cm. System-level and satellite-level DVI reduce this to 4.33 and 4.07 cm, respectively, corresponding to reductions of approximately 67% and 69%. Satellite-level DVI provides a further reduction of approximately 6%. Thus, the method improves PPP accuracy during short-term SSR outages, with modest satellite-level gains that vary by station, component, and outage window.

## 1. Introduction

In recent years, real-time precise point positioning (RT-PPP) has been widely used in geohazard monitoring, transportation, and precision agriculture [1,2,3]. Meanwhile, emerging applications, including autonomous driving, unmanned aerial vehicles, and low-altitude aircraft, have further broadened the scope of real-time high-precision positioning and imposed more stringent requirements on positioning services in terms of accuracy, continuity, availability, and reliability [4,5]. Precise orbit, clock, and various bias corrections required for RT-PPP are typically disseminated as state space representation (SSR) messages via Networked Transport of RTCM via Internet Protocol (NTRIP), where RTCM denotes the Radio Technical Commission for Maritime Services. By combining the received SSR corrections with broadcast ephemerides, users can reconstruct real-time precise satellite orbits and clocks, thereby supporting centimeter-level real-time positioning. Therefore, the accuracy, availability, timeliness, and transmission continuity of SSR products directly affect the positioning performance and service quality of RT-PPP.

Satellite orbit and clock corrections are external inputs to PPP processing, and their residual errors propagate into estimated parameters such as receiver coordinates, receiver clock offsets, and ambiguities. Previous studies have used post-processed precise products as references to assess real-time SSR products in terms of availability, transmission latency, orbit and clock accuracy, and PPP performance, revealing quality differences among analysis centers, Global Navigation Satellite System (GNSS) constellations, and orbit types [6,7,8,9]. To support quality monitoring and integrity in real-time applications, network observation residuals, anomaly detection algorithms, quality indicators, and range-domain protection levels have been used to identify product anomalies and support satellite exclusion or downweighting [10,11,12,13,14,15]. Whereas residual-based anomaly detection and adaptive or robust adjustment respond to current observations, this study uses historical product extrapolation errors to derive prior variance information, which can complement these quality-control measures.

Processing latency and communication outages increase the data age of the SSR corrections available to users. When the last valid SSR correction message is retained during an outage, orbit and clock extrapolation errors generally increase with data age. Previous studies have addressed this problem through product prediction and stochastic model optimization. Polynomial, time-series, and deep-learning models have been used to predict SSR corrections [16,17,18]. Related work has investigated satellite clock prediction [19], precise orbit extrapolation [20], and prediction of Galileo broadcast orbit errors [21]. For stochastic model optimization, Shu et al. [22] used ultra-rapid predicted orbits and added age-dependent clock extrapolation-error variance to the original observation variance. Mu et al. [23] used orbit and clock signal-in-space range error (SISRE) to optimize PPP and time-differenced carrier phase (TDCP) stochastic models, combining PPP with ambiguity resolution (PPP-AR) and TDCP switching to improve positioning under SSR latency. Product prediction improves orbit and clock estimates during outages, whereas stochastic model adjustment characterizes residual error uncertainty; the two approaches can be combined.

These studies provide a basis for using extrapolation errors to refine PPP stochastic models. For PPP using the last valid SSR message, it remains necessary to clarify how historical error samples should be used to construct fixed statistical error models. Their use in characterizing prior additional variances on independent dates also requires evaluation. Whether inter-satellite error differences provide additional positioning benefits requires comparison of system-level and satellite-level models using the same extrapolated products and variance-inflation procedure.

Building on observation variance inflation, this study proposes a multi-GNSS PPP stochastic model refinement method based on historical mean-square statistics of additional SISRE to mitigate positioning degradation during short-term SSR outages. We first characterize real-time SSR stream outages, select 10 min as the primary evaluation window, and assess continuously updated real-time orbit and clock accuracy. We then analyze age-dependent extrapolation errors and differences among constellations and satellites to construct statistical error models. The method is intended for real-time PPP users whose processing software supports SSR corrections and adjustment of the observation stochastic model. Historical models are constructed offline and queried online using satellite information and extrapolation age to determine dynamic observation weights. Finally, we compare models constructed from independent dates to assess their cross-day consistency. Four-constellation simulated kinematic PPP experiments evaluate positioning performance and quantify the additional benefit of satellite-level over system-level modeling.

## 2. Methodology

This section presents the reconstruction of precise satellite orbits and clocks from SSR correction messages, the extrapolation of SSR corrections during short-term outages, and PPP stochastic model refinement using historical extrapolation-error statistics.

### 2.1. Reconstruction of Precise Orbits and Clocks from SSR Corrections

Real-time SSR orbit and clock products provide corrections to satellite orbits and clocks derived from broadcast ephemerides. Users first calculate the broadcast values and then apply the SSR corrections to reconstruct precise satellite orbits and clocks at the processing epoch [24]. SSR orbit messages generally contain radial (R), along-track (A), and cross-track (C) corrections and their rates.

Let $t_{0}$ denote the reference epoch of an SSR orbit correction message and t the processing epoch. The orbit correction components at the reference epoch and their rates in the RAC frame are written as

(1) $$\delta O=\left[\begin{array}{c} \delta O_{r}\\ \delta O_{a}\\ \delta O_{c} \end{array}\right],\dot{\delta }O=\left[\begin{array}{c} \dot{\delta }O_{r}\\ \dot{\delta }O_{a}\\ \dot{\delta }O_{c} \end{array}\right]$$

where ${\delta O}_{r},{\delta O}_{a},{\delta O}_{c}$ are the radial, along-track, and cross-track orbit corrections, respectively, in m, and $\dot{\delta }O_{r},\dot{\delta }O_{a},\dot{\delta }O_{c}$ are their corresponding rates in m/s.

The RAC orbit correction at epoch t is then

(2) $$\delta O(t)=\delta O+\dot{\delta }O(t-t_{0})$$

GNSS positioning is usually performed in the Earth-centered, Earth-fixed (ECEF) frame; therefore, the orbit correction must be transformed from the local RAC frame into ECEF coordinates. Let r and $\dot{r}$ denote the satellite position and velocity vectors calculated from broadcast ephemerides. The RAC unit vectors are defined as

(3) $$\begin{array}{l} e_{a}=\frac{\dot{r}}{∥\dot{r}∥}\\ e_{c}=\frac{r\times \dot{r}}{∥r\times \dot{r}∥}\\ e_{r}=e_{a}\times e_{c} \end{array}$$

Here, $e_{a},e_{c},e_{r}$ denote the along-track, cross-track, and radial unit vectors, respectively, forming a right-handed orthonormal basis. The satellite position correction in ECEF coordinates is

(4) $$\delta X(t)=\left[\begin{array}{ccc} e_{r} & e_{a} & e_{c} \end{array}\right]\delta O(t)$$

The corrected satellite position is then

(5) $$X_{orbit}(t)=X_{brdc}(t)-\delta X(t)$$

where $X_{orbit}(t)$ is the satellite position after applying the SSR orbit correction and $X_{brdc}(t)$ is the position calculated from broadcast ephemerides.

The precise satellite clock offset is obtained from the broadcast clock offset and SSR clock correction as

(6) $$t_{sat}(t)=t_{brdc}(t)+\frac{\Delta C(t)}{c}$$

where $t_{sat}(t)$ and $t_{brdc}(t)$ are the SSR-corrected and broadcast satellite clock offsets, respectively, both in seconds (s); ΔC(t) is the range-equivalent SSR clock correction in meters (m); and c is the speed of light in vacuum.

The SSR clock correction is generally evaluated from a second-order polynomial referenced to epoch $t_{0}$:

(7) $$\Delta C(t)=C_{0}+C_{1}(t-t_{0})+C_{2}{\left(t-t_{0}\right)}^{2}+\Delta C_{hr}$$

where $C_{0},C_{1},C_{2}$ are the SSR clock polynomial coefficients, with units of m, m/s, and m/s^2^, respectively, and ${\Delta C}_{hr}$ is the high-rate SSR clock correction in m.

### 2.2. Extrapolation of SSR Corrections During Data Outages

During a short-term outage of a real-time stream, users cannot obtain new SSR correction messages. A direct approach is to retain the last valid SSR message received before the outage and use its orbit and clock corrections to reconstruct satellite orbits and clocks during the interruption. This approach is referred to here as SSR correction extrapolation.

Let $t_{L}$ be the reference epoch of the last valid SSR correction message received before the outage and t the processing epoch during the outage. The corresponding data age is

(8) $$\tau =t-t_{L}$$

The SSR message referenced to $t_{L}$ is retained throughout the outage. Let ${\delta O}_{L}$ and $\dot{\delta }O_{L}$ denote its RAC orbit corrections and rates, respectively. The RAC orbit correction at processing epoch t is

(9) $$\delta O_{L}(t)=\delta O_{L}+\dot{\delta }O_{L}(t-t_{L})$$

The RAC unit vectors are determined from the broadcast satellite position and velocity at epoch t, yielding the ECEF satellite position correction:

(10) $$\delta X_{L}(t)=\left[\begin{array}{ccc} e_{r}(t) & e_{a}(t) & e_{c}(t) \end{array}\right]\delta O_{L}(t)$$

The extrapolated satellite position is therefore

(11) $$X_{orbit}^{ext}(t)=X_{brdc}(t)-\delta X_{L}(t)$$

For the satellite clock, let $C_{0,L},C_{1,L},C_{2,L}$ be the polynomial coefficients in the last valid SSR clock correction message. The clock correction at epoch t is

(12) $$\Delta C_{L}(t)=C_{0,L}+C_{1,L}(t-t_{L})+C_{2,L}{\left(t-t_{L}\right)}^{2}+\Delta C_{hr,L}$$

where ${\Delta C}_{hr,L}$ is the last valid high-rate clock correction obtained before the outage. It is held constant throughout the simulated outage window and added directly to the extrapolated clock polynomial. This hold-last-value treatment is an extrapolation assumption; unmodeled changes in the high-rate term contribute to the additional clock extrapolation error. The satellite clock offset reconstructed using the extrapolated SSR correction is then

(13) $$t_{sat}^{ext}(t)=t_{brdc}(t)+\frac{\Delta C_{L}(t)}{c}$$

### 2.3. PPP Stochastic Model Refinement Accounting for Orbit and Clock Extrapolation Errors

Historical mean-square statistics of additional SISRE are used to construct system-level and satellite-level age-dependent statistical error models. These models refine the PPP observation stochastic model through direct variance inflation (DVI). During offline modeling, continuously updated SSR messages provide reference orbits and clocks at the same epoch. Subtracting these reference values from the extrapolated values gives signed additional extrapolation errors.

For satellite s, let i=(d,k) index the kth extrapolation event on date d, and let τ denote extrapolation age. The radial, along-track, and cross-track components of the additional orbit extrapolation error are denoted by ${\Delta R}_{s,i}(\tau )$, ${\Delta A}_{s,i}(\tau )$, and ${\Delta C}_{s,i}(\tau )$, respectively, in centimeters. The additional clock extrapolation error is ${\Delta t}_{s,i}(\tau )$, in seconds. A separate receiver clock offset is estimated for each GNSS constellation, allowing the corresponding parameter to absorb a clock offset common to its satellites. For each event and extrapolation age, the common clock component is estimated as the median additional clock extrapolation error across valid satellites in the constellation. This median is then subtracted from each satellite’s error. The valid-satellite set is determined by product availability rather than visibility at an individual station. The remaining clock error is converted into a range error:

(14) $$\delta \rho_{s,i}(\tau )=100c\left[\Delta t_{s,i}(\tau )-\underset{j\in g(s)}{median}(\Delta t_{j,i}(\tau ))\right]$$

where ${\delta \rho }_{s,i}(\tau )$ is the range-equivalent additional clock extrapolation error after removal of the constellation-common component, in cm; g(s) identifies the GNSS constellation of satellite s; c is the speed of light in vacuum; and the factor 100 converts m to cm.

Following the combined orbit and clock SISRE formulation of Montenbruck et al. [25], the orbit errors and the range-equivalent clock error after common-component removal are combined to define the squared additional SISRE as

(15) $$e_{s,i}^{2}(\tau )={\left[\omega_{R,s}\Delta R_{s,i}(\tau )-\delta \rho_{s,i}(\tau )\right]}^{2}+\omega_{AC,s}^{2}[\Delta A_{s,i}^{2}(\tau )+\Delta C_{s,i}^{2}(\tau )]$$

where $\omega_{R,s}$ and $\omega_{AC,s}^{2}$ are geometric weighting factors for the statistical contributions of radial and along-track/cross-track orbit errors, respectively, to user line-of-sight range errors. Their values depend primarily on satellite altitude. The constellation- and orbit-type-dependent SISRE geometric weights follow previous studies [26,27], with the numerical values taken from Kazmierski et al. [28], as listed in Table 1. MEO, IGSO, and GEO denote medium Earth, inclined geosynchronous, and geostationary orbits, respectively. The signed difference between the radial orbit error and the range-equivalent clock error is squared, preserving their cross term.

To give different training days equal weight, the daily mean of the squared additional SISRE for satellite s on date d at age τ is first calculated as

(16) $${\bar{e}}_{s,d}^{2}(\tau )=\frac{1}{K_{s,d}(\tau )}\sum_{k=1}^{K_{s,d}(\tau )}e_{s,(d,k)}^{2}(\tau )$$

where $e_{s,(d,k)}^{2}(\tau )$ is the squared additional SISRE calculated using Equation (15) for satellite s, extrapolation event k on date d, and age τ; $K_{s,d}(\tau )$ is the corresponding number of valid extrapolation events. Averaging the daily values with equal weights across valid training days gives the satellite-level mean-square statistic of additional SISRE:

(17) $$M_{s}(\tau )=\frac{1}{D_{s}(\tau )}\sum_{d=1}^{D_{s}(\tau )}{\bar{e}}_{s,d}^{2}(\tau )$$

where $D_{s}(\tau )$ is the number of valid training days for satellite s at age τ.

For each training day and extrapolation age, the daily means of squared additional SISRE are averaged with equal weights across valid satellites within the constellation. The resulting daily values are then averaged with equal weights across valid training days to obtain the system-level mean-square statistic of additional SISRE:

(18) $$M_{g}(\tau )=\frac{1}{D_{g}(\tau )}\sum_{d=1}^{D_{g}(\tau )}\left[\frac{1}{S_{g,d}(\tau )}\sum_{s\in g}{\bar{e}}_{s,d}^{2}(\tau )\right]$$

where $S_{g,d}(\tau )$ is the number of valid satellites in constellation g on date d at age τ, and $D_{g}(\tau )$ is the corresponding number of valid training days. The age-dependent statistics $M_{s}(\tau )$ and $M_{g}(\tau )$ form the satellite-level and system-level statistical error models, respectively.

When $D_{s}(\tau )=0$, satellite s has no valid training samples of its own at that age. The valid statistic of its constellation is used as a fallback:

(19) $$M_{s}(\tau )=M_{g(s)}(\tau )$$

The model outputs $M_{s}(\tau )$ and $M_{g}(\tau )$ are converted from cm^2^ to m^2^ and used as equivalent additional variances in the PPP observation stochastic model:

(20) $$q_{\mathrm{add},s}^{2}(\tau )=10^{-4}\left\{\begin{array}{c} M_{s}(\tau ),\mathrm{Satellite}\text{-}\mathrm{level}\;\mathrm{DVI}\\ M_{g(s)}(\tau ),\mathrm{System}\text{-}\mathrm{level}\;\mathrm{DVI} \end{array}\right.$$

where $q_{add,s}^{2}(\tau )$ is in m^2^. The two DVI schemes use the same variance-inflation procedure and differ only in whether satellite-level or system-level error statistics are used.

At PPP processing epoch t, let $\tau_{s,t}$ be the extrapolation age of satellite s, and let $\sigma_{P,s,t,0}^{2}$ and $\sigma_{L,s,t,0}^{2}$ be the baseline variances of the ionosphere-free pseudorange and carrier-phase combinations, respectively, both in m^2^. Orbit and clock errors produce a common range effect in the dual-frequency observations of a satellite, which is retained in the ionosphere-free combination. Under a diagonal stochastic-model approximation, the same equivalent additional variance is therefore added to the respective baseline variances of the two observation types:

(21) $$\left\{\begin{array}{c} \sigma_{P,s,t,DVI}^{2}=\sigma_{P,s,t,0}^{2}+q_{\mathrm{add},s}^{2}(\tau_{s,t})\\ \sigma_{L,s,t,DVI}^{2}=\sigma_{L,s,t,0}^{2}+q_{\mathrm{add},s}^{2}(\tau_{s,t}) \end{array}\right.$$

The corresponding observation weights are

(22) $$\left\{\begin{array}{c} \omega_{P,s,t}=\frac{1}{\sigma_{P,s,t,DVI}^{2}}\\ \omega_{L,s,t}=\frac{1}{\sigma_{L,s,t,DVI}^{2}} \end{array}\right.$$

During online processing, the user queries the stored statistical error model using the satellite constellation, identifier, and extrapolation age. Equations (20)–(22) then determine the equivalent additional variance and observation weights. Continuously updated SSR reference products are needed only for offline modeling and experimental evaluation, not during online outages.

The equivalent additional variance is derived from historical mean-square statistics of additional SISRE and approximates the combined range effect of orbit and clock extrapolation errors. This statistic includes error variability and any contribution from bias. It is not the variance of range errors along the actual line of sight at a specific station. Equation (21) adds the equivalent variance only to the diagonal entries of the observation covariance matrix. It does not explicitly model covariance between baseline and additional extrapolation errors or temporal correlations in the extrapolation errors. It also omits correlations induced by satellite product errors between pseudorange and carrier-phase observations and between observations at different stations. The method therefore refines a diagonal stochastic model using historical error statistics rather than describing the full product-error covariance structure.

## 3. Experimental Data and Processing Strategies

### 3.1. Data Sources and Training–Test Split

Real-time SSR data from the APM, CNE, and Wuhan University (WHU) analysis centers were received and archived using BKG Ntrip Client (BNC) 2.12 [29]. The dataset covers 30 days, from day of year (DOY) 032 to 061 of 2026. Concurrent data from all three centers were used to characterize short-term SSR stream outages. WHU data were additionally used to assess product availability, continuously updated real-time orbit and clock accuracy, and short-term extrapolation errors, as well as to construct statistical error models, compare them across dates, and validate positioning performance.

The WHU data were randomly divided by date into non-overlapping training and test sets to construct and evaluate the system-level and satellite-level statistical error models. The 15 training days were DOY 032, 036, 037, 039, 042, 044, 046, 047, 049, 051, 052, 056, 057, 059, and 061. The 15 test days were DOY 033, 034, 035, 038, 040, 041, 043, 045, 048, 050, 053, 054, 055, 058, and 060. DOY 035, 048, and 058 from the test set were selected for the simulated kinematic PPP experiments described in Section 3.4.

Both statistical error models used for PPP positioning were constructed exclusively from the training set and remained fixed during the experiments. Test data were not used to determine or adjust these statistics. Reference models constructed from the test set were used only for retrospective cross-day comparisons and did not determine the additional variances used in PPP processing.

### 3.2. SSR Stream Outage Statistics and Real-Time Product Accuracy Assessment

Outage characteristics and product availability were determined from the received and archived SSR data. The nominal interval between SSR reference epochs was 5 s for all three centers. A reference epoch lacking an orbit or clock data block was classified as an outage epoch. Consecutive outage epochs within a day were grouped into one event, and a single missing epoch also counted as an event. Event duration was calculated as the number of missing epochs multiplied by 5 s. Events were counted separately for each analysis center. An outage affecting multiple constellations simultaneously at one center was counted once rather than once per constellation. For availability statistics, a satellite was considered available at an epoch only when both valid orbit and clock data blocks were present; daily availability was then calculated for each constellation.

WHU SSR corrections from the SSRC00WHU0 mountpoint were combined with multi-GNSS broadcast ephemerides provided by the International GNSS Service (IGS) to reconstruct real-time precise orbits and clocks following the method in Section 2.1. Final precise products from the Center for Orbit Determination in Europe (CODE) served as the reference [30]. Both product sets used GPS time (GPST), and both orbit products used the IGc20 reference frame. As the orbits from both centers were referenced to the satellite center of mass (CoM), no additional satellite antenna offset correction was applied during reconstruction or accuracy assessment. The SSR orbit and clock update intervals were both 5 s, whereas the CODE orbit and clock sampling intervals were 5 min and 30 s, respectively. Comparisons used only common satellites and epochs to avoid interpolation errors; neither interpolation nor zero-filling was applied.

For orbit accuracy assessment, differences between the reconstructed WHU orbits and CODE final orbits were calculated at common epochs and projected from ECEF coordinates onto the RAC frame. For each satellite, day, and component, samples outside the mean ± three sample standard deviations were excluded before calculating the daily root mean square (RMS) error. For each satellite and component, daily RMS values exceeding three times the median of the daily series were then removed. The remaining daily RMS values were averaged arithmetically, and the satellite averages were given equal weight to obtain constellation- and orbit-type-level results.

Clock accuracy was assessed using a second-differencing procedure to remove common datum offsets between WHU and CODE clocks [31]. Clock differences were first calculated for common satellites and epochs and prescreened using the median absolute deviation (MAD) for each satellite and day. Within each constellation and epoch, valid satellite differences were then screened robustly, and their median was subtracted from each satellite difference to remove the common clock datum offset. For each satellite’s daily residual series, samples with absolute values greater than three times the median absolute residual were excluded before calculating the daily standard deviation (STD). Daily STD values exceeding three times the median of that satellite’s daily STD series were subsequently removed, and the retained values were averaged. Equal averaging of the satellite-level mean STDs yielded the constellation- and orbit-type-level results.

### 3.3. Orbit and Clock Extrapolation Experiments and Cross-Day Model Comparisons

The 30-day dataset was used to construct 10 min simulated SSR outage windows beginning at minute 45 of every hour. This produced 24 scheduled windows per day and 720 scenarios in total, each containing 120 extrapolation epochs at 5 s intervals. Consecutive window start times were separated by 1 h, so the windows did not overlap. SSR corrections were extrapolated using the method in Section 2.2, and additional extrapolation errors were calculated as defined in Section 2.3.

For cross-day comparisons, satellite-level and system-level statistical error models were constructed separately from the 15 training days and the 15 test days following the uniform statistical criteria specified in Section 2.3.

The square root of the model’s additional SISRE mean-square statistic defines the error scale used for comparison. The satellite-level and system-level error scales are

$$E_{s}(\tau )=\sqrt{M_{s}(\tau )},E_{g}(\tau )=\sqrt{M_{g}(\tau )}$$

where $E_{s}(\tau )$ and $E_{g}(\tau )$ are in cm. Comparisons covered 0–10 min at 5 s age increments. Satellite-level comparisons used the same satellites and age nodes for which both sets had satellite-specific statistics; system-level comparisons used the same constellations and age nodes. The RMS of paired error-scale differences quantified the overall discrepancy between the two model sets.

To determine whether multi-day aggregation masked day-to-day variability, the fixed training model was also compared separately with each test-day model. The RMS of the error-scale differences was calculated for each test day.

### 3.4. Multi-GNSS Simulated Kinematic PPP Experiments

Multi-GNSS simulated kinematic PPP experiments used GPS, Galileo, the third-generation BeiDou Navigation Satellite System (BDS-3), and GLONASS observations from nine static IGS stations. The stations were AMC4, BRST, DLF1, GRAZ, ONSA, PNGM, POTS, SPT0, and TOW2. The observation dates were DOY 035, 048, and 058 of 2026. The experiments assessed the positioning benefit of stochastic model refinement during short-term SSR product outages. Three simulated 10 min outages began at 07:45, 14:45, and 21:45 GPST each day, giving nine outage scenarios. Station coordinates were estimated independently at each epoch to evaluate positioning performance in simulated kinematic mode.

Four positioning schemes were compared. Scheme 1 used precise orbits and clocks reconstructed from continuously updated SSR messages as the uninterrupted reference. Scheme 2 used products extrapolated from the last valid SSR message before the outage, retaining the original PPP stochastic model without additional downweighting. Schemes 3 and 4 used the same extrapolated products as Scheme 2 and refined the observation stochastic model through DVI as described in Section 2.3, using system-level and satellite-level statistical error models, respectively. They are referred to below as the system-level and satellite-level DVI schemes.

Schemes 3 and 4 used the training-set models specified in Section 3.1. Comparisons with Scheme 2 evaluated the positioning benefit of DVI based on historical error statistics. Comparing Scheme 4 with Scheme 3 quantified the additional benefit of satellite-level over system-level modeling.

Each scenario was processed independently for 7 h. The simulated outage occurred within the final processing hour, and processing continued to the end of that hour. Before the outage, all schemes used continuously updated SSR products without additional downweighting and shared the same initialization and baseline processing strategies. During the outage, products and weights were applied according to the schemes above. The processing interval was 5 s, giving 120 epochs per station in a complete outage window. Table 2 summarizes the processing strategies and parameter settings.

Epoch-wise positioning errors in the east (E), north (N), and up (U) components were calculated relative to the IGS daily station coordinates. Valid solutions from the four schemes were matched by date, outage window, station, and epoch, leaving 9695 common valid station–epoch records per scheme. This was 25 fewer than the theoretical total of 9720; all missing records were at PNGM, while the other eight stations had complete coverage. The nine windows were aligned by extrapolation age. At each age, mean absolute errors (MAEs) in E, N, and U were calculated across the common valid samples. Squared component errors were also pooled over 0–5, 5–10, and 0–10 min and averaged before taking the square root to obtain overall RMS errors. The 3D RMS was calculated as the square root of the sum of the squared component RMS values. RMS reduction rates were calculated relative to the corresponding reference scheme; positive values indicate improvement and negative values indicate degradation.

## 4. Results and Discussion

Using the data and processing strategies in Section 3, this section first examines SSR stream outages, product availability, and continuously updated real-time product quality. It then analyzes orbit and clock extrapolation errors introduced by short-term outages and the cross-day consistency of their statistical models. Finally, it compares the positioning benefits of system-level and satellite-level DVI in multi-GNSS simulated kinematic PPP.

### 4.1. Short-Term SSR Stream Outages and Product Availability

#### 4.1.1. Short-Term SSR Stream Outage Characteristics

**Figure 1:**
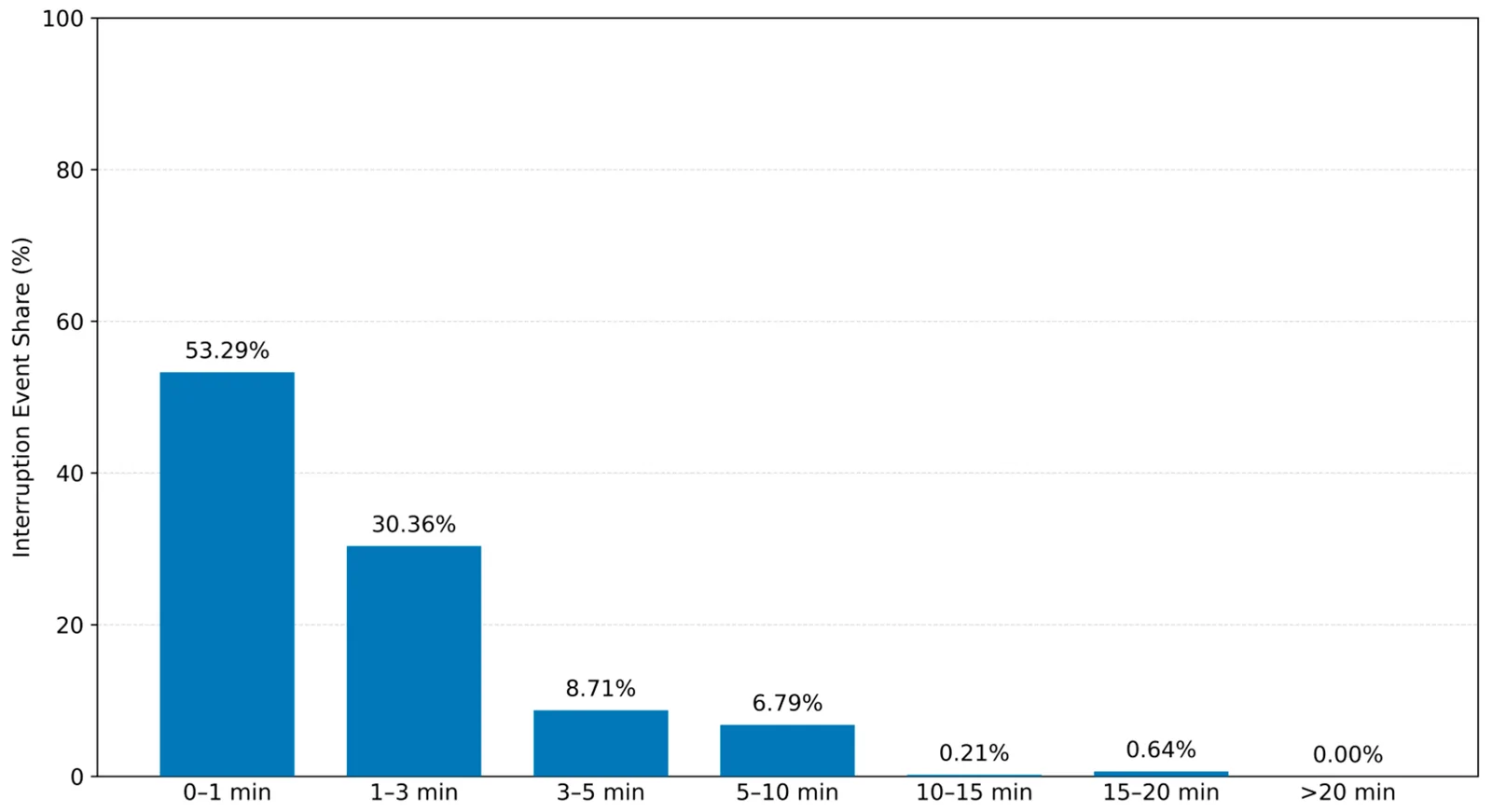
Duration distribution of real-time SSR stream outages at the APM, CNE, and WHU analysis centers.

Figure 1 shows the duration distribution of all outage events from the three analysis centers. A total of 471 events were detected over 30 days. Events lasting 0–1 min accounted for 53.29%, and the cumulative proportions lasting no more than 5 and 10 min were 92.36% and 99.15%, respectively, indicating that short outages predominated. Events lasting 10–15 and 15–20 min accounted for 0.21% and 0.64%, respectively. No outage longer than 20 min was recorded.

Table 3 compares the outage distributions among centers. APM, CNE, and WHU had 262, 116, and 93 events, respectively. Outages lasting 0–1 min predominated at APM and WHU, accounting for 66.03% and 51.61%, respectively. By contrast, the 3–5 and 5–10 min categories each accounted for 23.28% of CNE events, exceeding the corresponding proportions at APM and WHU and indicating a higher proportion of intermediate-duration outages during the study period. Nevertheless, events no longer than 10 min accounted for 99.62%, 99.14%, and 97.85% at APM, CNE, and WHU, respectively. Thus, outages within 10 min predominated at all three centers. These statistics describe the streams received during the analyzed 30-day period; their long-term representativeness requires longer observations.

#### 4.1.2. Availability of WHU Real-Time SSR Orbit and Clock Products

**Figure 2:**
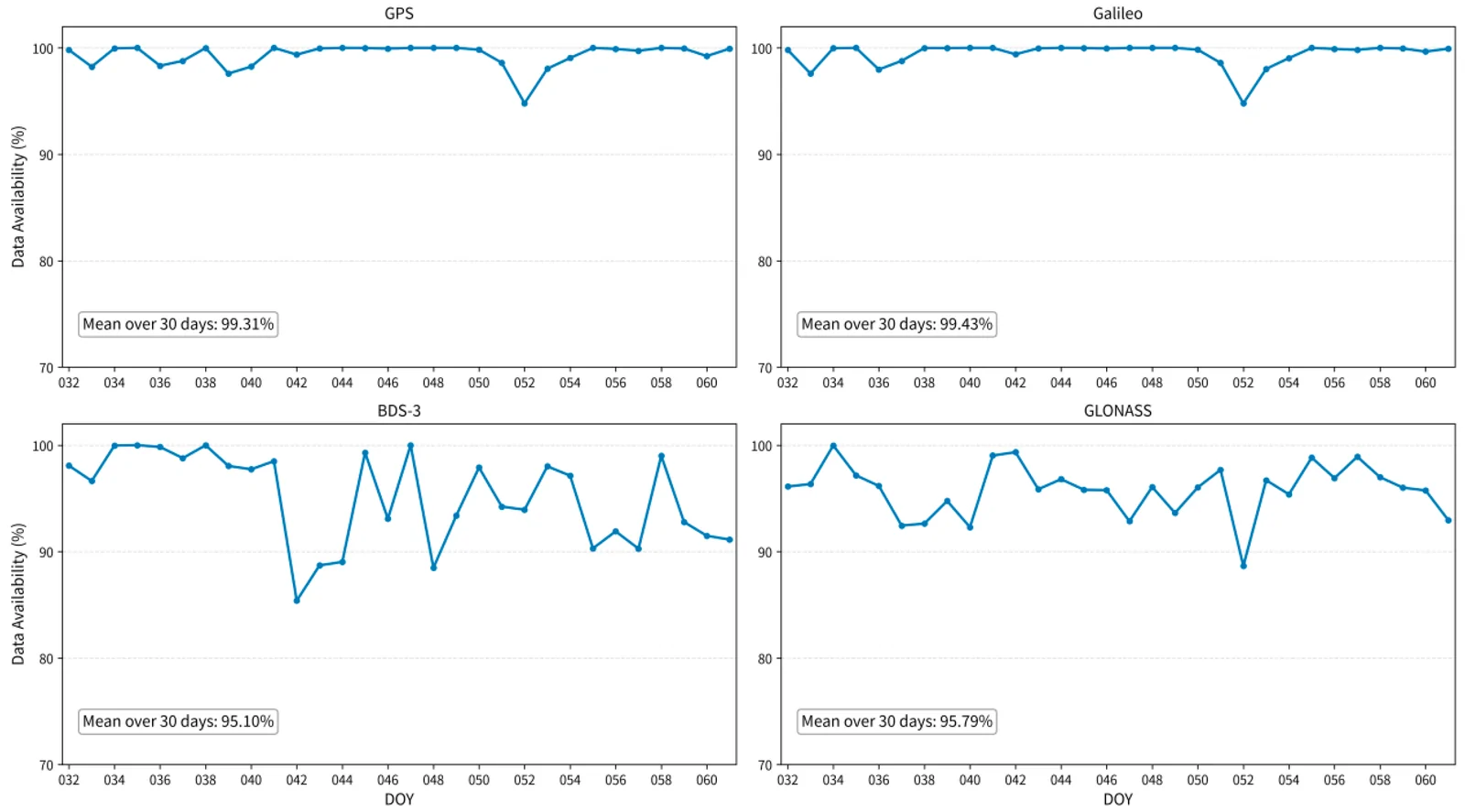
Daily mean availability of WHU multi-GNSS real-time SSR orbit and clock products.

Figure 2 shows the daily mean availability of WHU real-time SSR orbit and clock products for the four constellations. GPS and Galileo were the most stable, with 30-day mean availabilities of 99.31% and 99.43%, respectively. Both declined noticeably only on DOY 052, to 94.79% and 94.78%; availability exceeded 95% on all other days. BDS-3 and GLONASS had lower 30-day means of 95.10% and 95.79% and greater day-to-day variability. BDS-3 availability was below 90% on DOY 042, 043, 044, and 048, reaching a minimum of 85.38%. GLONASS reached its minimum of 88.64% on DOY 052 and remained above 93% on all other days.

### 4.2. Accuracy Assessment of WHU Real-Time SSR Orbit and Clock Products

Table 4 lists the satellite counts and valid sample counts retained after matching common satellites and epochs and screening outliers following the procedures in Section 3.2. Samples are counted as satellite–epoch records: one valid error record for one satellite at one epoch constitutes one sample. The final R, A, and C sample counts differ because outlier and cross-day screening were performed separately for each component.

**Figure 3:**
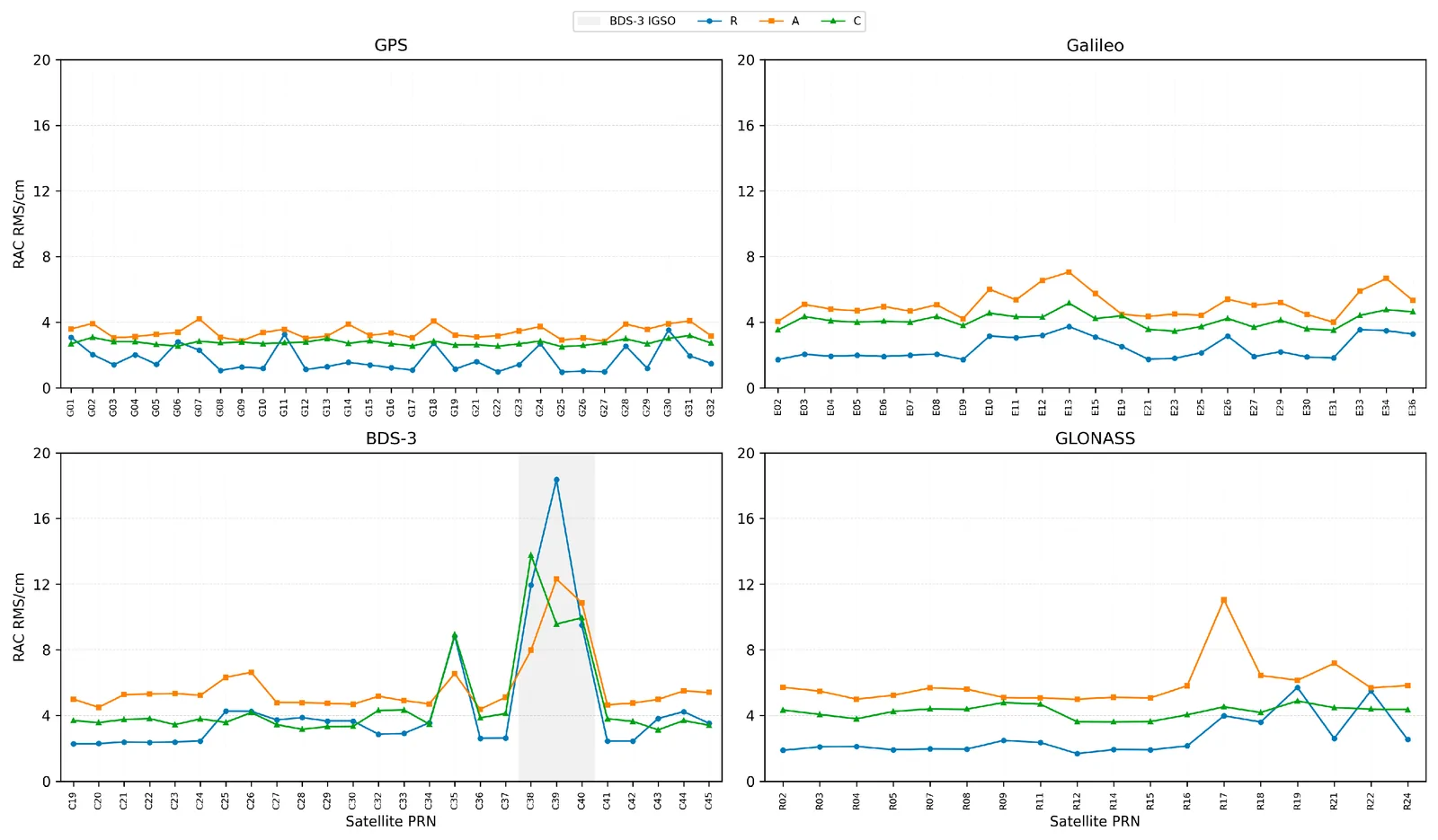
Satellite-wise mean RMS orbit errors in the R, A, and C components for WHU multi-GNSS real-time products.

Figure 3 and Table 5 report mean RMS orbit errors in the R, A, and C components, obtained using the averaging procedure in Section 3.2. Real-time orbit accuracy differs among constellations and orbit types. The GPS real-time orbits agree closely with the CODE final orbits, with RMS values of 1.73, 3.39, and 2.76 cm in R, A, and C, respectively. Galileo and GLONASS have RMS values below 6 cm in all components, with relatively larger along-track errors. BDS-3 MEO satellites have corresponding RMS values of 3.36, 5.16, and 3.90 cm, comparable to Galileo and GLONASS. BDS-3 IGSO satellites, however, have substantially larger RMS values of 13.26, 10.38, and 11.09 cm. Previous studies suggest that BDS IGSO orbit accuracy may be affected by observation geometry, solar radiation pressure modeling, and satellite attitude [32,33,34,35].

**Figure 4:**
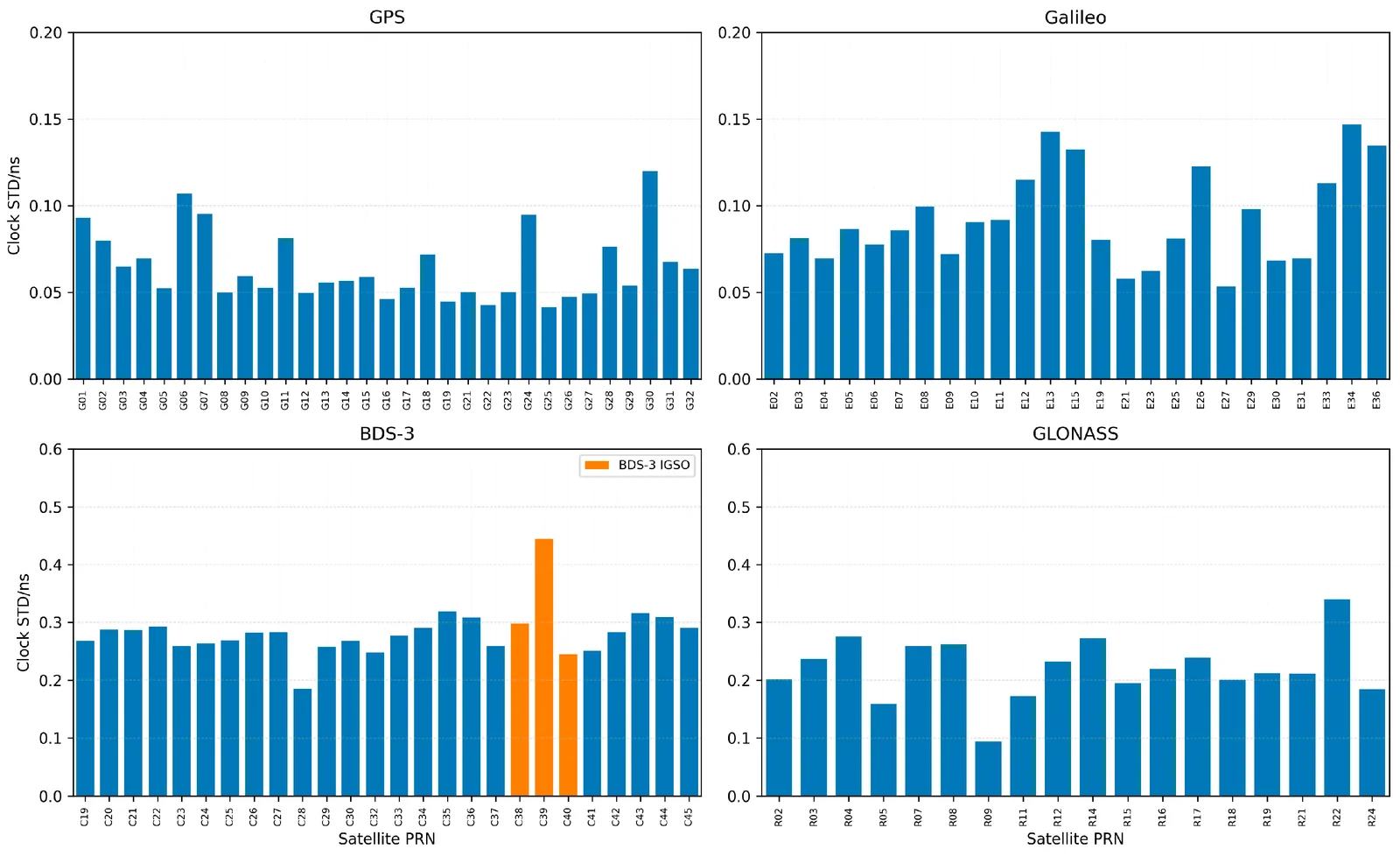
Satellite-wise mean clock residual STDs for WHU multi-GNSS real-time products.

Figure 4 and Table 6 show clock residual STDs by constellation and orbit type. GPS and Galileo have relatively small mean STDs of 0.064 and 0.092 ns, respectively, indicating close agreement with CODE final clocks. GLONASS has a larger mean STD of 0.220 ns. BDS-3 clock STDs are higher overall, with mean values of 0.276 and 0.329 ns for MEO and IGSO satellites, respectively. The mean STD for IGSO satellites is approximately 19.2% higher than that for MEO satellites. Mean clock residual STDs increase in the order GPS, Galileo, GLONASS, BDS-3 MEO, and BDS-3 IGSO, reflecting constellation- and orbit-type-dependent differences in real-time clock quality.

To assess the persistence of differences identified by multi-day aggregation, Table 7 summarizes the medians and ranges of daily accuracy metrics. Median daily orbit RMS values are higher for BDS-3 IGSO than for MEO in all three components, and their ranges are wider. This indicates larger orbit errors and greater absolute day-to-day variability for IGSO satellites. GPS daily clock STD ranges from 0.048 to 0.083 ns, compared with 0.120–0.612 ns for BDS-3 MEO and 0.177–0.809 ns for BDS-3 IGSO. Both BDS-3 orbit classes therefore show larger clock STDs and wider absolute day-to-day ranges.

Same-day comparisons show higher orbit RMS values for BDS-3 IGSO than for MEO in all three components on all 30 days. GPS clock STD is lower than that of Galileo on 29 days, whereas GLONASS clock STD exceeds those of both systems on all 30 days. BDS-3 IGSO clock STD exceeds that of MEO on 21 days. These results support the main differences identified by the multi-day summaries. However, the relative ordering of clock STDs for GPS versus Galileo and BDS-3 IGSO versus MEO varies among dates.

Overall, continuously updated WHU real-time SSR orbit and clock products show clear differences among constellations and orbit types. These results describe agreement with CODE final precise products, rather than the additional extrapolation errors caused by short-term outages. The next section evaluates these additional errors relative to products reconstructed from continuously updated SSR messages at the same epochs, examining age dependence and differences among constellations and satellites.

### 4.3. Orbit and Clock Extrapolation Errors During Short-Term Outages

#### 4.3.1. Orbit Extrapolation Error Characteristics

**Figure 5:**
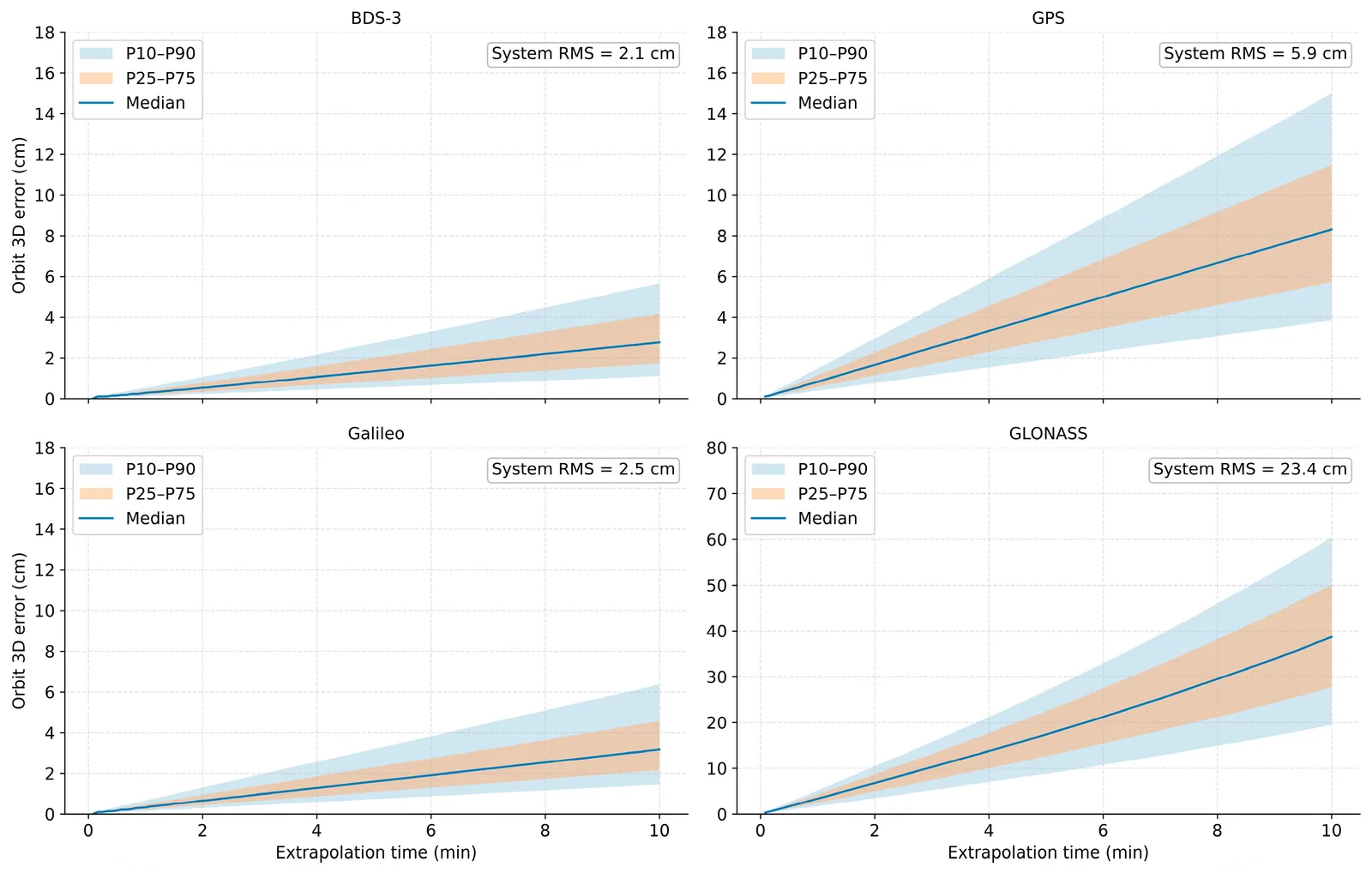
Age-dependent percentile intervals of 3D orbit extrapolation errors under SSR correction extrapolation. Note: At each age, percentiles are calculated by pooling valid 3D orbit extrapolation-error samples across dates, simulated windows, and satellites. The numbers of valid extrapolation events range from 712 to 718 for BDS-3, 712 to 718 for GPS, 712 to 717 for Galileo, and 712 to 718 for GLONASS. At 600 s, all four constellations have 714 valid events.

Figure 5 shows the median, P25–P75 interval, and P10–P90 interval of 3D orbit extrapolation errors as functions of extrapolation age for the four constellations. Medians generally increase and both percentile intervals widen with age, indicating increases in error magnitude and dispersion among satellites and outage windows. BDS-3 and Galileo show relatively gradual error growth. GPS has larger errors and a wider distribution, whereas GLONASS has substantially higher errors and larger absolute increases than the other constellations.

Table 8 reports the overall RMS 3D orbit extrapolation errors for different age intervals. For BDS-3, GPS, Galileo, and GLONASS, RMS errors increase from 1.0, 3.0, 1.3, and 11.0 cm over 0–5 min to 2.7, 7.9, 3.3, and 31.2 cm over 5–10 min, respectively. These are approximately 2.7, 2.6, 2.5, and 2.8 times the first-5-min values. All four constellations show clear age-dependent error accumulation, with GLONASS having the largest absolute errors in every interval. Although BDS-3 and Galileo retain lower error levels, their RMS errors also increase markedly in the second half of the window. The GPS RMS over 0–10 min is 5.9 cm, approximately 2.6 times the mean of the corresponding BDS-3 and Galileo values.

**Figure 6:**
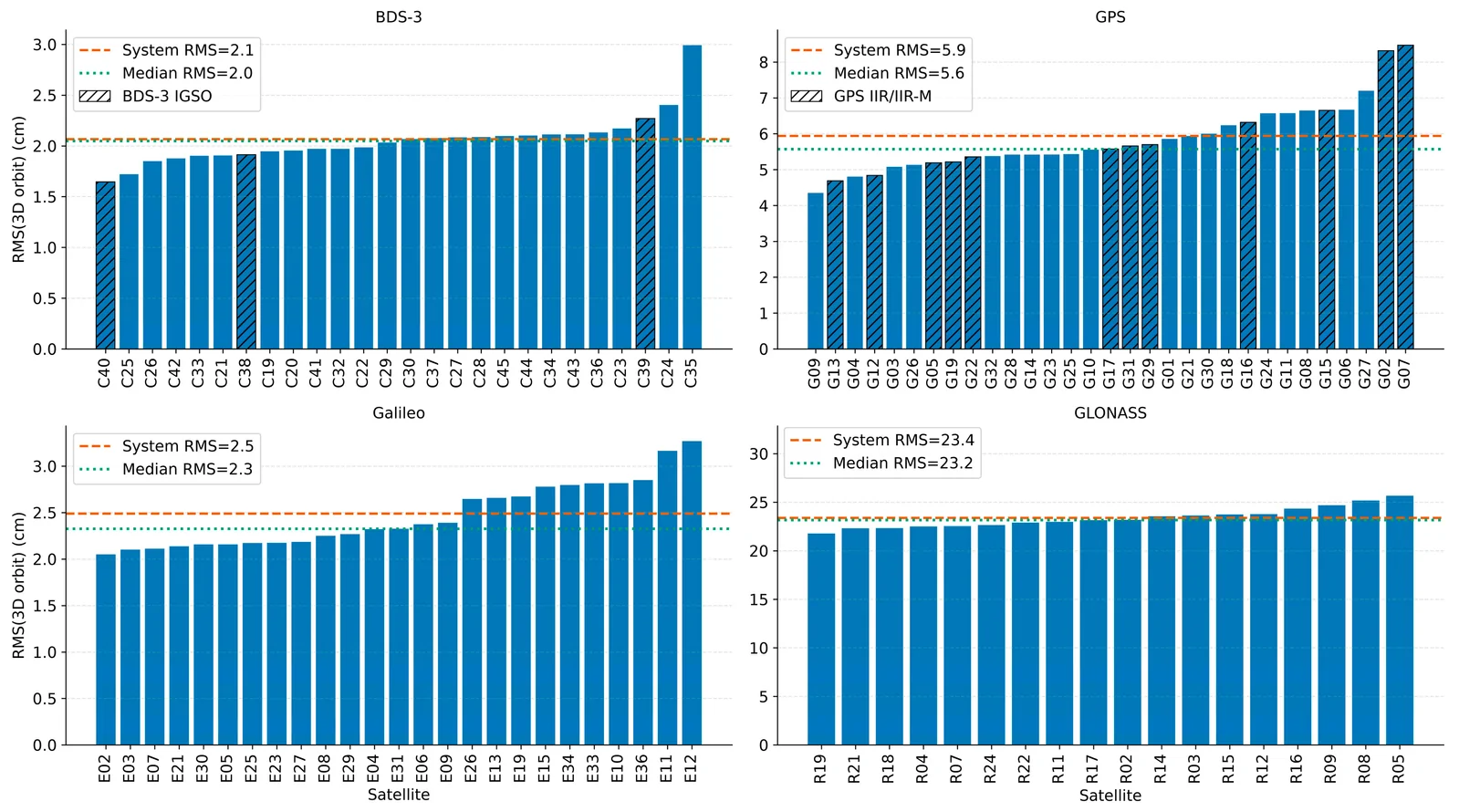
Satellite-wise RMS ranking of 3D orbit extrapolation errors over 0–10 min under SSR correction extrapolation.

Figure 6 ranks the satellite-wise RMS 3D orbit extrapolation errors over 0–10 min. Overall constellation RMS values for BDS-3, GPS, Galileo, and GLONASS are 2.1, 5.9, 2.5, and 23.4 cm, respectively, while the corresponding medians of satellite-wise RMS values are 2.0, 5.6, 2.3, and 23.2 cm. Their proximity indicates that the constellation-level results are comparable in magnitude to the errors of most satellites. Within-constellation differences nevertheless require examination of the satellite-wise distributions.

BDS-3 satellite RMS values are relatively concentrated, ranging from 1.65 to 2.99 cm. Galileo values range from 2.05 to 3.27 cm, with E11 and E12 relatively high at 3.16 and 3.27 cm. GPS has more pronounced inter-satellite differences, ranging from 4.35 to 8.47 cm; G02 and G07 reach 8.31 and 8.47 cm. All GLONASS satellites have relatively large errors, with RMS values of 21.79–25.67 cm and smaller relative inter-satellite differences than GPS. The larger GLONASS extrapolation errors therefore reflect elevated errors across most satellites in that constellation.

In summary, orbit extrapolation errors generally increase with age and differ markedly among constellations, with appreciable inter-satellite differences in some constellations.

#### 4.3.2. Clock Extrapolation Error Characteristics

**Figure 7:**
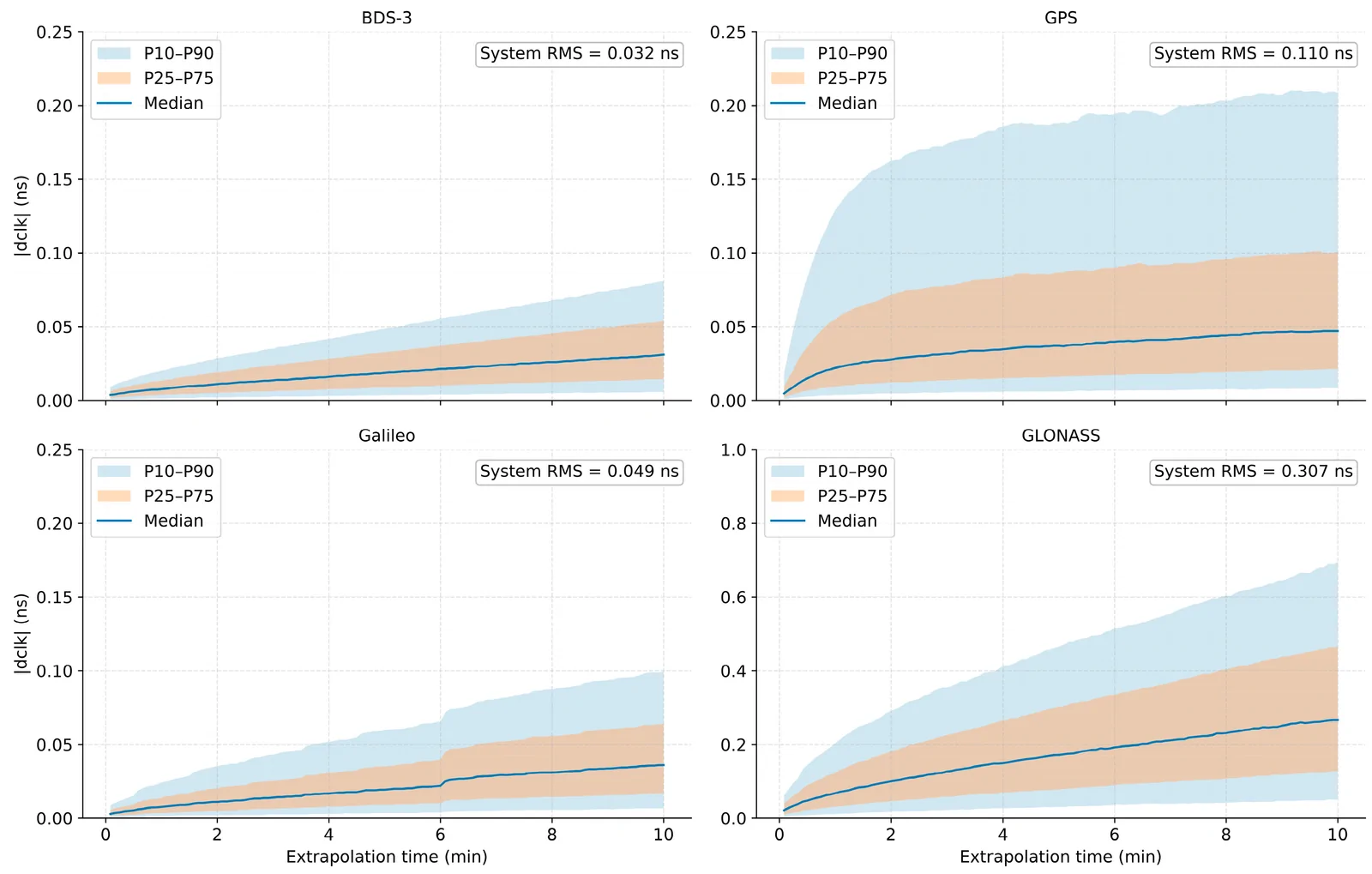
Age-dependent percentile intervals of absolute clock extrapolation errors under SSR correction extrapolation. Note: At each age, percentiles are calculated by pooling valid absolute clock extrapolation-error samples across dates, simulated windows, and satellites. The numbers of valid events range from 709 to 715 for BDS-3, 709 to 715 for GPS, 709 to 714 for Galileo, and 709 to 715 for GLONASS. At 600 s, all four constellations have 711 valid events.

Figure 7 shows the median and P25–P75 and P10–P90 intervals of absolute clock extrapolation errors as functions of age. Across all constellations, medians generally increase and percentile intervals widen with age, indicating both larger errors and greater dispersion. BDS-3 and Galileo maintain relatively low errors throughout the window. For GPS, the P10–P90 interval widens rapidly at small ages while the median grows more gradually, indicating that some large-error samples strongly affect the upper tail. GLONASS has substantially larger medians and wider percentile intervals than the other constellations, both increasing with age.

Table 9 reports overall RMS clock extrapolation errors for different age intervals. Over 0–10 min, the values are 0.032, 0.110, 0.049, and 0.307 ns for BDS-3, GPS, Galileo, and GLONASS, respectively. BDS-3 has the smallest errors and GLONASS the largest. For all four constellations, RMS errors over 5–10 min exceed those over 0–5 min. The 5–10 min values are 0.041, 0.124, 0.061, and 0.374 ns, respectively. These are approximately 2.1, 1.3, 2.0, and 1.7 times the corresponding 0–5 min values. Clock extrapolation errors therefore show clear age-dependent accumulation, with differences among constellations in both error magnitude and the increase with extrapolation age.

**Figure 8:**
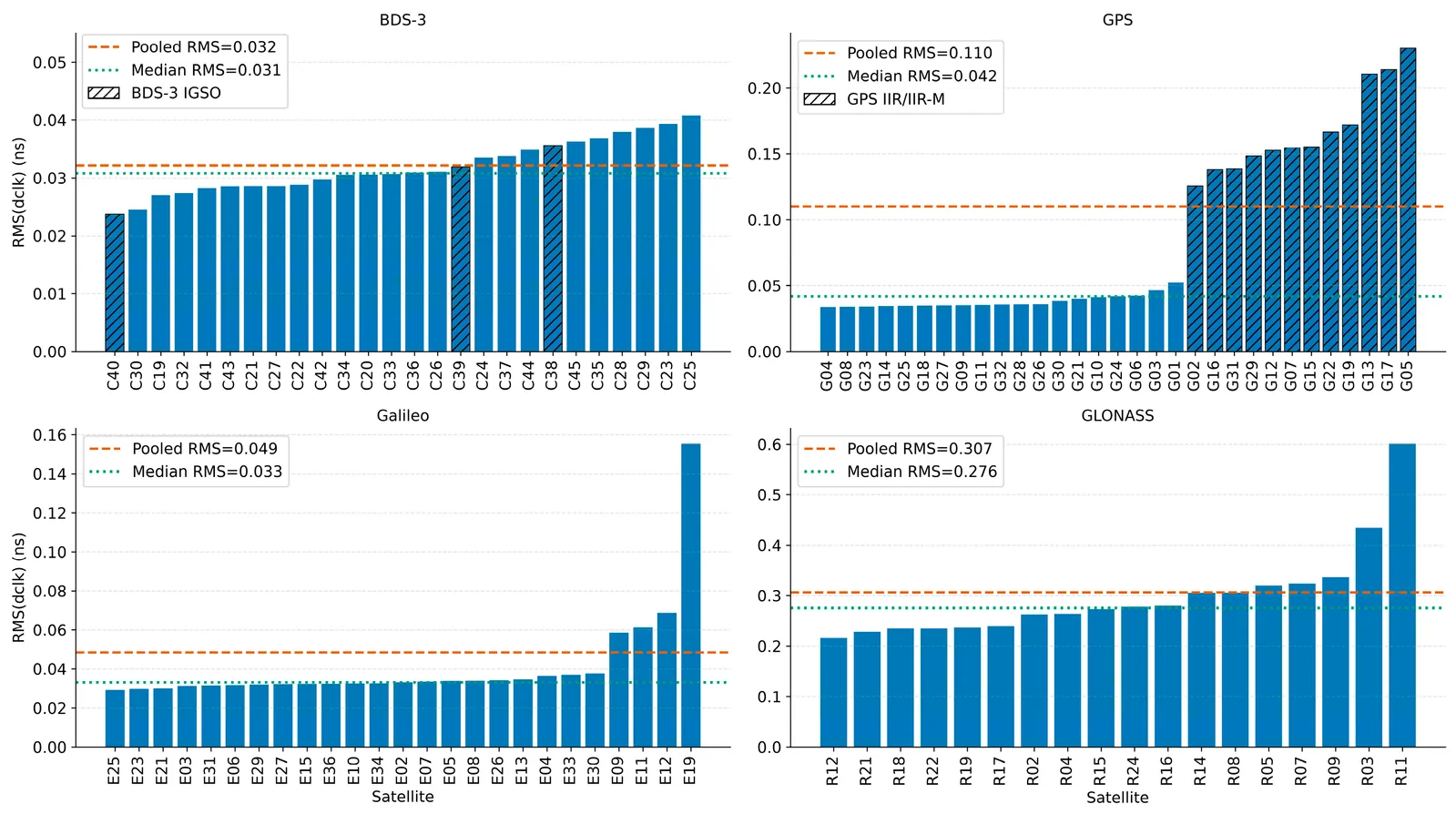
Satellite-wise RMS ranking of clock extrapolation errors over 0–10 min under SSR correction extrapolation.

Figure 8 ranks satellite-wise RMS clock extrapolation errors over 0–10 min. BDS-3 values range from 0.024 to 0.041 ns, with a median of 0.031 ns close to the overall RMS of 0.032 ns, indicating relatively small inter-satellite differences. GPS exhibits distinct satellite groups. The 12 IIR/IIR-M satellites G02, G05, G07, G12, G13, G15, G16, G17, G19, G22, G29, and G31 have a mean RMS of 0.167 ns, approximately 4.4 times the mean RMS of 0.038 ns for the remaining GPS satellites. This is consistent with previous reports of relatively weaker short-term clock stability for IIR/IIR-M satellites [36,37]. The median GPS satellite RMS is 0.042 ns, well below the overall RMS of 0.110 ns, indicating the substantial influence of the high-error satellite group.

Most Galileo satellites have low errors, but E19 reaches an RMS of 0.155 ns, approximately 3.2 times the overall value of 0.049 ns. This may be related to previously reported nonstationarity and changes in short-term stability of the E19 clock series [38]; the specific cause requires verification using clock series and stability metrics from the experimental period. The GLONASS satellite RMS median of 0.276 ns is close to the overall RMS of 0.307 ns, showing that its elevated errors are not confined to a few satellites. R03 and R11 reach 0.434 and 0.601 ns, respectively. These findings are broadly consistent with the relatively weaker short-term frequency stability reported for traditional GLONASS onboard clocks, although SSR estimation noise and satellite clock operating states may also affect extrapolation errors [37,39]. These satellite groups and individual-satellite patterns characterize the study period and WHU products; their persistence at other times requires longer datasets.

Clock extrapolation errors are age-dependent and differ among constellations and satellites. Together with the orbit results, these characteristics motivate the construction of system-level and satellite-level age-dependent statistical error models.

### 4.4. Cross-Day Consistency of Statistical Extrapolation-Error Models

**Figure 9:**
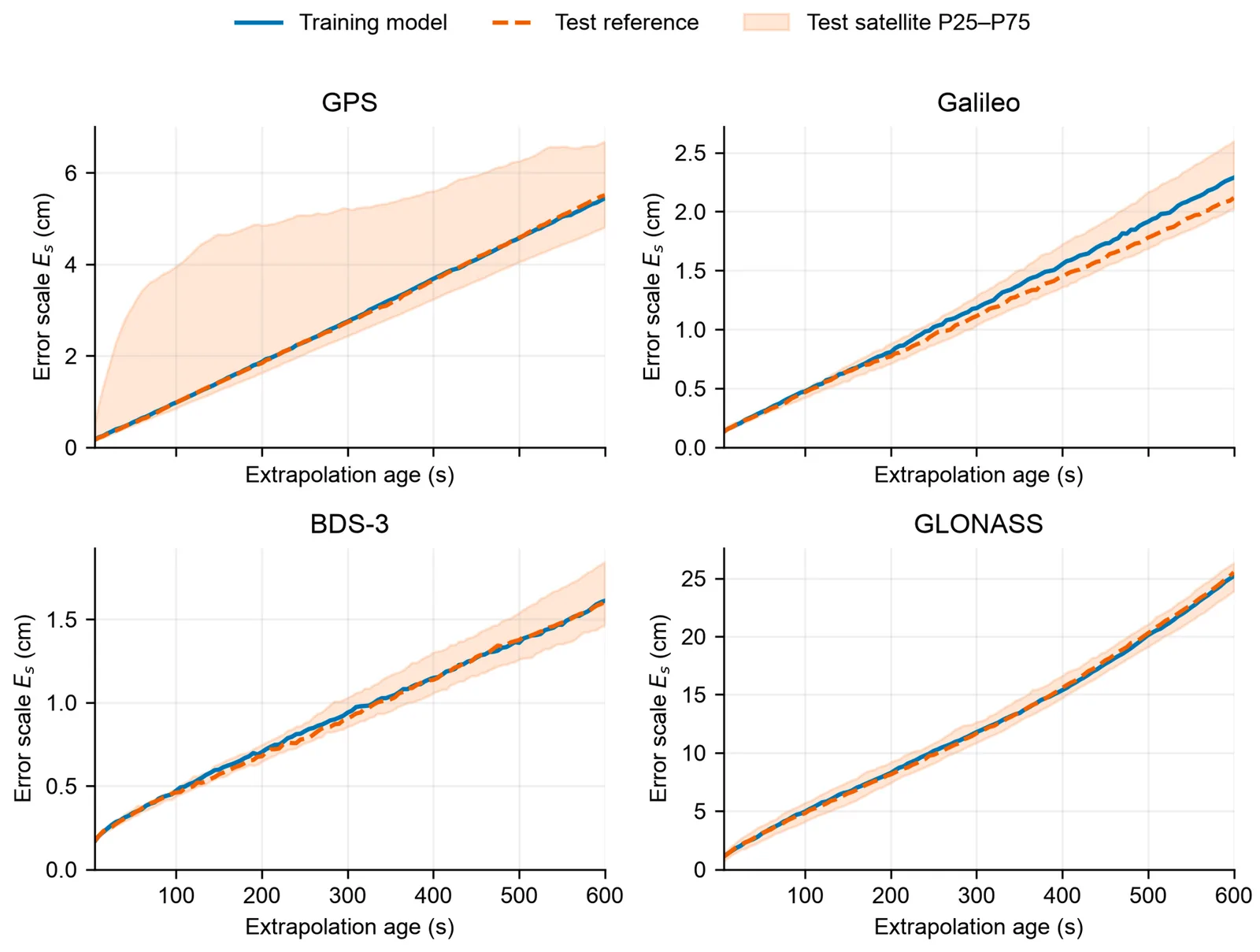
Age-dependent satellite-level additional SISRE error scales of the training model and test reference model.

Figure 9 shows the age-dependent satellite-level error scales for each constellation in the training and test sets. Solid and dashed lines denote the respective medians across satellites valid in both sets at each age. Shading shows the test-set P25–P75 interval, representing inter-satellite variability within a constellation. For each constellation, the training and test median curves are generally close and increase with extrapolation age. At 10 min, training-set medians for GPS, Galileo, BDS-3, and GLONASS are 5.45, 2.29, 1.61, and 25.21 cm, respectively, compared with test-set values of 5.51, 2.12, 1.61, and 25.51 cm. The corresponding absolute differences are 0.07, 0.17, 0.01, and 0.30 cm. The GPS percentile interval is wide relative to its median, consistent with the higher clock extrapolation errors of IIR/IIR-M satellites in Section 4.3.2. GLONASS retains a high error scale in both sets, consistent with its larger orbit and clock extrapolation errors and their combined effect in the range domain.

**Figure 10:**
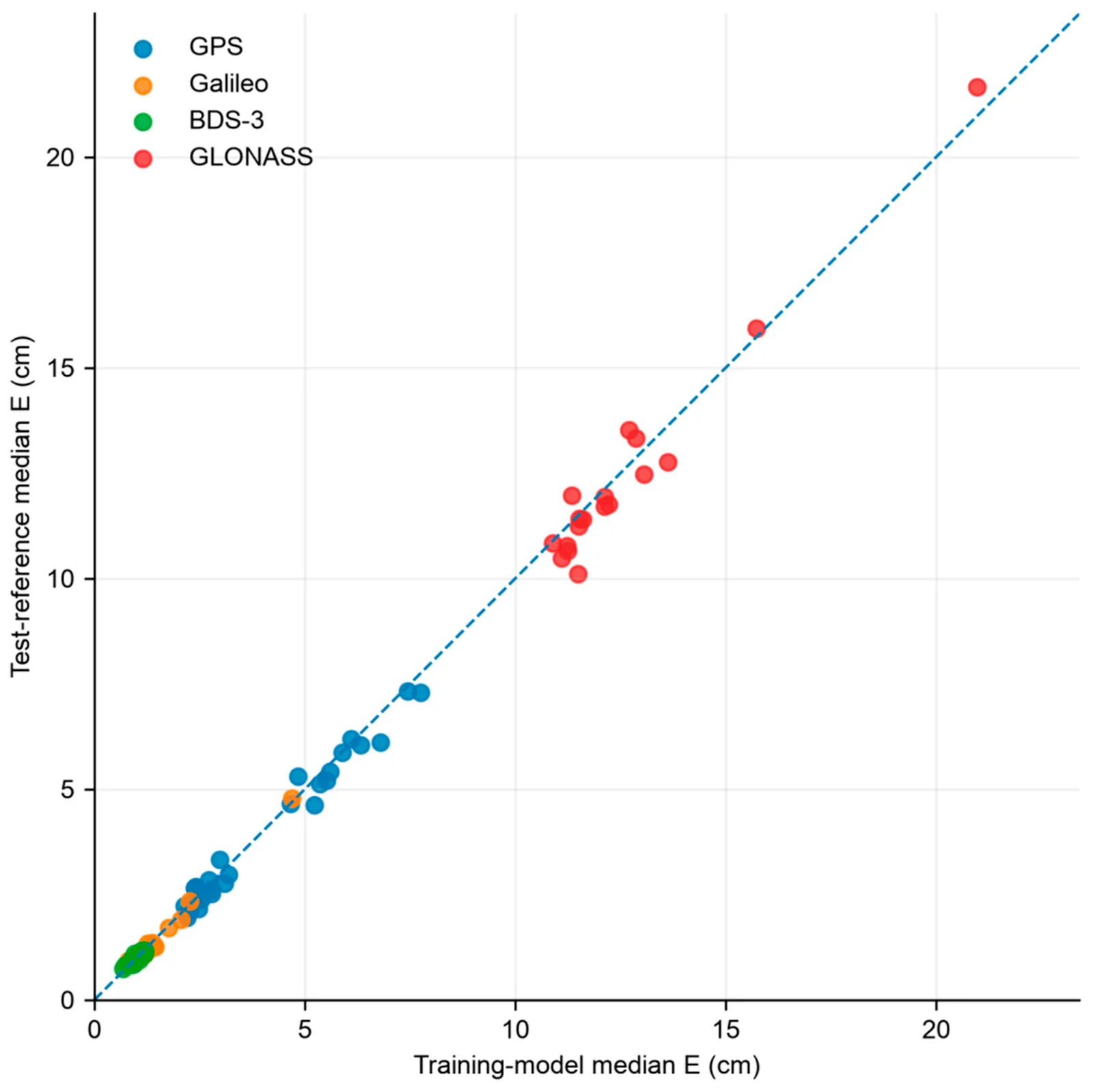
Satellite-level consistency between the training model and test reference model.The blue dashed line represents the 1:1 reference line (y = x).

Figure 10 compares satellite-wise median error scales to further examine consistency between the training model and test reference model. Each point represents one satellite. Its horizontal and vertical coordinates are the median error scales of that satellite in the training and test reference models, respectively, calculated over their common valid age nodes within 0–10 min. The reference line is y=x. Most points lie close to this line, indicating similar overall satellite error scales between the two date sets. Most BDS-3 and Galileo points lie in the region of lower error scales and near the reference line. Their relatively low error scales are therefore maintained in both models. The 12 GPS IIR/IIR-M satellites generally have larger error scales than other GPS satellite types in both sets. This agrees with the clock-error grouping in Section 4.3.2 and indicates a degree of cross-day repeatability. GLONASS satellites R03 and R11 have high error scales in both models. These satellites also have the two largest RMS clock extrapolation errors among GLONASS satellites in Section 4.3.2.

Table 10 quantifies differences between the training model and test reference model using the RMS of their additional SISRE error-scale differences over 0–10 min. Across the four constellations, these RMS values range from 0.085 to 0.687 cm at the satellite level and from 0.009 to 0.181 cm at the system level. Together with Figure 9 and Figure 10, these results support overall agreement between the two models. BDS-3 and Galileo show relatively small absolute error-scale differences in both the satellite-level and system-level comparisons. GLONASS has a larger satellite-level RMS of error-scale differences, consistent with deviations from the reference line in Figure 10. Because error scales differ in magnitude among constellations, these absolute RMS values alone should not be used to rank relative consistency. Satellite-level and system-level statistics represent different levels of aggregation, so these RMS values cannot determine which DVI scheme yields better PPP accuracy.

The fixed training model was compared separately with each of the 15 test-day models. The medians of the daily RMS values of satellite-level error-scale differences are 1.064, 0.297, 0.220, and 2.102 cm for GPS, Galileo, BDS-3, and GLONASS, respectively. The corresponding ranges are 0.932–1.278, 0.244–0.451, 0.147–0.248, and 1.856–2.845 cm. BDS-3 shows smaller daily absolute differences within a narrower range, whereas GPS and GLONASS show larger daily discrepancies. These daily discrepancies exceed those between the multi-day aggregated models. Agreement between multi-day models therefore does not imply equally close error scales between the historical model and every individual date.

The age-dependent and satellite-wise comparisons show that, within the study period and for WHU products, the training model and test reference model are broadly consistent in overall error scale, age-dependent growth, and the main GPS satellite grouping. This supports the use of historical extrapolation-error statistics as prior information for PPP stochastic model refinement. The next section evaluates the resulting positioning benefit through multi-GNSS simulated kinematic PPP experiments.

### 4.5. Multi-GNSS Simulated Kinematic PPP Performance During Short-Term Outages

**Figure 11:**
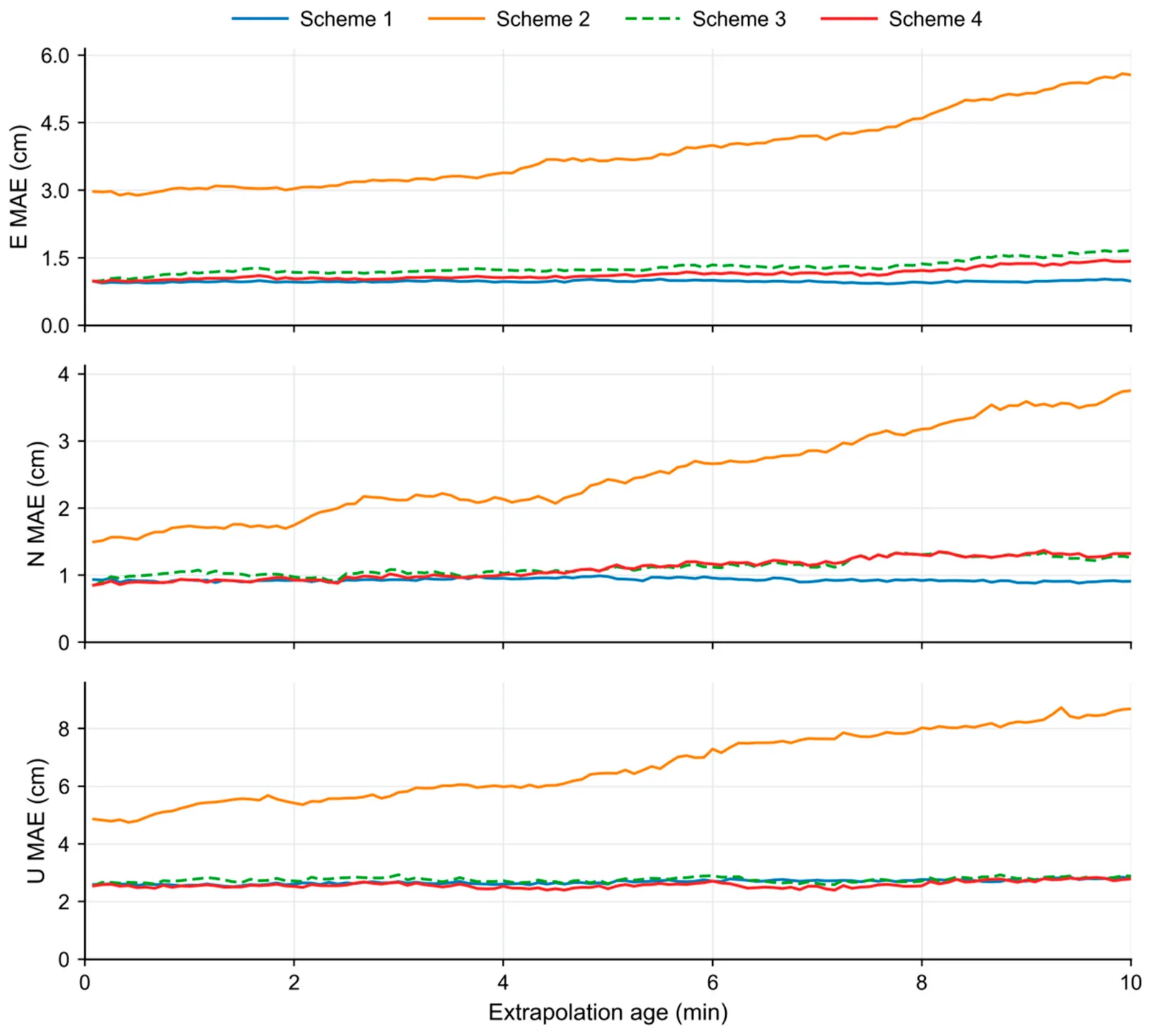
E-, N-, and U-component MAEs versus extrapolation age for the four schemes during simulated SSR outages.

Figure 11 shows E-, N-, and U-component MAEs for the four schemes across nine stations and nine simulated outage windows over three days, aligned by extrapolation age. The MAEs of Scheme 1, the continuous-SSR reference, remain generally stable. Scheme 2, extrapolation without additional downweighting, shows increasing errors in all three components, with a larger absolute increase in U. Both DVI schemes markedly suppress this growth, keeping the U-component MAE broadly stable. Their curves are generally close. Compared with system-level DVI (Scheme 3), satellite-level DVI (Scheme 4) has lower E-component MAE at most age nodes and slightly lower U-component MAE overall, whereas their N-component curves cross.

Table 11 compares overall positioning accuracy and between-station variability by reporting RMS errors over different age intervals and the median and range of station-level 3D RMS over 0–10 min. Scheme 2 has an overall 3D RMS of 13.26 cm, which decreases to 4.33 cm with system-level DVI and 4.07 cm with satellite-level DVI, reductions of 67.33% and 69.29%, respectively. RMS errors also decrease in all three components. Both DVI schemes improve positioning with extrapolated products, although the 3D RMS of Scheme 4 remains above the 3.83 cm of the continuous-SSR reference.

Relative to system-level DVI, satellite-level DVI further reduces the E-, N-, U-, and 3D RMS over 0–10 min by 6.63%, 3.79%, 6.17%, and 5.98%, respectively. The corresponding 3D RMS reductions are 7.47% over 0–5 min and 4.71% over 5–10 min. Thus, the relative improvement is smaller in the second half of the window. All component RMS values decrease in both intervals, although the N-component reduction over 5–10 min is only 0.58%. The additional benefit of satellite-level modeling therefore varies with component and interval.

Station-level statistics over 0–10 min show that the median 3D RMS decreases from 9.78 cm for Scheme 2 to 3.92 and 3.65 cm for system-level and satellite-level DVI, respectively. The maximum station-level 3D RMS also decreases from 23.33 cm to 7.01 and 6.87 cm. Both DVI schemes therefore reduce the median and maximum station-level errors, with satellite-level DVI yielding slightly lower values.

**Figure 12:**
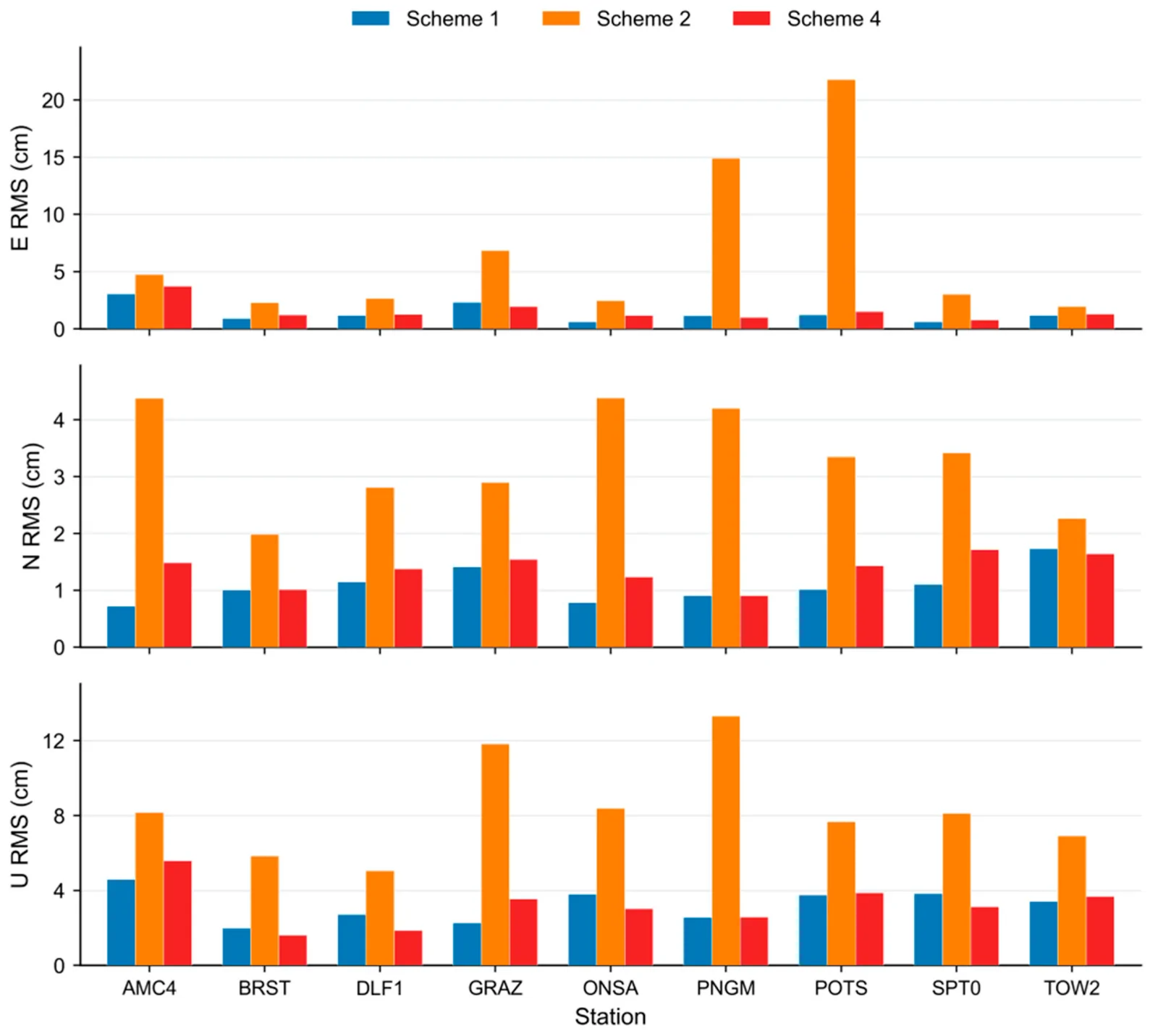
Station-wise RMS positioning errors for the continuous-SSR reference, extrapolation without additional downweighting, and satellite-level DVI.

Figure 12 examines how the overall improvement varies among stations and components. It compares station-wise E-, N-, and U-component RMS values over 0–10 min for Schemes 1, 2 and 4. Relative to Scheme 2, Scheme 4 improves all three components at all nine stations. The reduction ranges are 21.73–93.30% for E, 27.54–78.40% for N, and 31.61–80.64% for U, showing substantial between-station variability. Differences between Scheme 4 and the continuous-SSR reference also vary with station and component.

**Figure 13:**
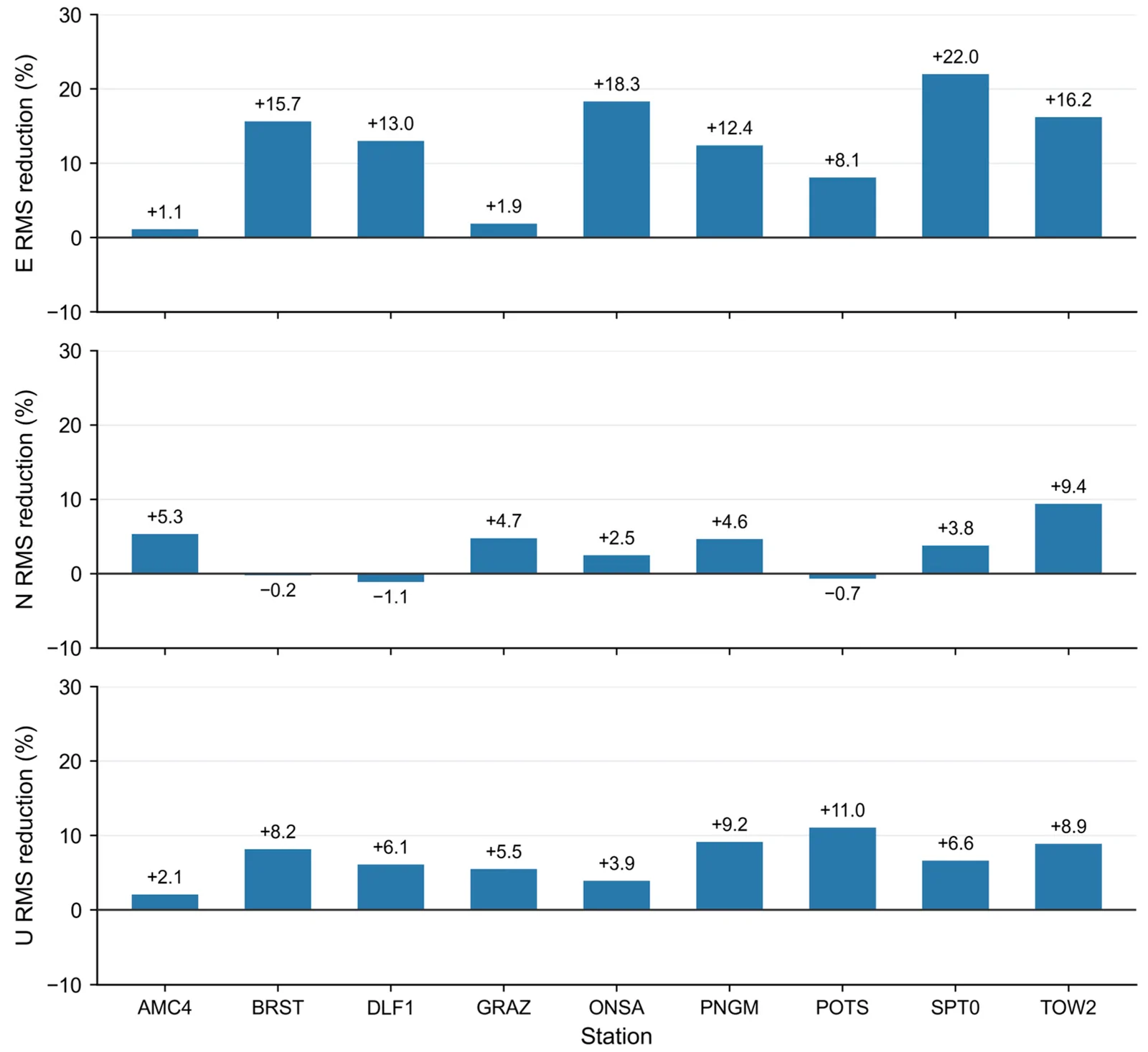
Station-wise RMS positioning error reductions achieved by satellite-level DVI relative to system-level DVI.

Figure 13 quantifies the additional benefit of satellite-level modeling through station-wise RMS reductions relative to system-level DVI. RMS errors decrease in both E and U at all nine stations. SPT0 has the largest E-component reduction, 22.00%, and POTS has the largest U-component reduction, 11.04%. The N component improves at six stations but worsens at BRST, DLF1, and POTS, where RMS increases by 0.22%, 1.13%, and 0.67%, respectively. Thus, the benefit of satellite-level modeling is not uniform across stations and components.

Table 12 compares 3D RMS values across the nine windows to examine the two DVI schemes under different outage scenarios. Satellite-level DVI reduces 3D RMS in seven windows by 3.68–20.17% relative to system-level DVI. Relative to the same baseline, 3D RMS instead increases by 0.85% and 1.01% in the 07:45 windows on DOY 048 and 058, respectively. The satellite-level scheme’s window-wise 3D RMS ranges from 2.44 to 7.70 cm, with the maximum in the 14:45 window on DOY 048. Satellite-level modeling therefore reduces 3D RMS in most windows, but its additional benefit depends on the outage scenario.

The age-dependent positioning errors and the overall, station-wise, and window-wise statistics show that DVI based on historical extrapolation-error statistics improves multi-GNSS simulated kinematic PPP accuracy during simulated short-term SSR outages. System-level and satellite-level DVI yield similar overall accuracy. Finer satellite-level modeling provides a modest overall gain, but its additional benefit varies with station, component, and outage scenario.

## 5. Conclusions

To mitigate PPP accuracy degradation during short-term SSR outages, this study constructs age-dependent statistical error models from historical mean-square statistics of additional SISRE and refines the PPP observation stochastic model through direct variance inflation. System-level and satellite-level modeling are compared in terms of positioning performance. The main conclusions are as follows:

(1) Real-time SSR stream outages are dominated by short-duration events. A total of 471 outages were detected at APM, CNE, and WHU over 30 days, of which 99.15% lasted no longer than 10 min. This supports the selection of 10 min as the primary window for evaluating extrapolation and positioning performance.
(2) Continuously updated real-time SSR orbit and clock quality differs among constellations and orbit types. Except for BDS-3 IGSO satellites, constellation-level orbit RMS values are generally below 6 cm in all RAC components. GPS and Galileo clock products agree more closely with the post-processed precise reference products, whereas GLONASS and BDS-3 have larger clock residual STDs. BDS-3 IGSO satellites show comparatively lower orbit and clock product quality.
(3) With SSR correction extrapolation, orbit and clock errors generally increase with data age and differ markedly among constellations and satellites. BDS-3 and Galileo have relatively small short-term errors. GPS shows pronounced inter-satellite differences, with IIR/IIR-M satellites having larger clock extrapolation errors than other GPS satellites. GLONASS has elevated errors across the constellation.
(4) Historical extrapolation-error statistics show a degree of cross-day repeatability. Models constructed from non-overlapping 15-day training and test sets show broadly similar error-scale growth with data age and preserve the main GPS satellite grouping. The RMS values of error-scale differences range from 0.085 to 0.687 cm at the satellite level and from 0.009 to 0.181 cm at the system level. These results support the use of historical models for prior error characterization. Daily discrepancies remain, however, so consistency between multi-day models does not imply identical error scales on every date.
(5) Direct variance inflation based on historical statistical error models improves multi-GNSS simulated kinematic PPP accuracy during short-term outages. Across nine windows over three days, system-level and satellite-level DVI reduce 3D RMS from 13.26 cm without additional downweighting to 4.33 and 4.07 cm, respectively. These correspond to reductions of 67.33% and 69.29%. Relative to system-level DVI, satellite-level DVI further reduces 3D RMS by 5.98%. It yields lower 3D RMS in seven of the nine windows, although its additional benefit varies with station, component, and window. The two schemes have similar overall accuracy, and the 3D RMS of the satellite-level DVI scheme remains higher than the 3.83 cm obtained with the continuous-SSR reference. Variance inflation therefore mitigates the positioning impact of extrapolation errors but does not fully compensate for degraded product accuracy.

PPP stochastic model refinement based on historical extrapolation-error statistics offers a feasible way to improve positioning during short-term SSR outages. It adjusts observation variances to mitigate error propagation without directly correcting the extrapolated orbits and clocks or recovering missing SSR information. These findings are based on simulated outages within the study period using WHU products. Further evaluation of model applicability requires longer datasets, products from other analysis centers, real SSR outages, and moving-receiver scenarios.

## Figures and Tables

**Table 1:** Weighting factors for the radial (R), along-track (A), and cross-track (C) components in SISRE calculation.

System (Type)	$\omega_{R}$	$\omega_{AC}^{2}$
GPS	0.98	1/49
Galileo	0.98	1/61
GLONASS	0.98	1/45
BDS-3 (MEO)	0.98	1/54
BDS-3 (IGSO, GEO)	0.99	1/126

**Table 2:** Data processing strategies and parameter settings for the simulated kinematic PPP experiments.

Parameter	Setting
GNSS constellations	GPS, Galileo, BDS-3, and GLONASS
Observations	Dual-frequency undifferenced pseudorange and carrier phase; GPS: L1/L2; Galileo: E1/E5a; BDS-3: B1I/B3I; GLONASS: L1/L2
Observation combination	Ionosphere-free combination
Positioning mode	Simulated kinematic PPP
Parameter estimation	Kalman filter
Sampling interval	5 s
Elevation cutoff	10°
Observation noise	A priori standard deviations of 0.30 m for pseudorange and 0.01 cycles for carrier phase
Elevation weighting	Elevation-dependent observation variances at elevations ≤30°; equal weighting above 30°
Receiver coordinates	Estimated independently at each epoch using a white-noise model
Receiver clocks	Estimated separately for each constellation using a white-noise model
Tropospheric delay	Saastamoinen model for the hydrostatic delay; random-walk estimation of the wet delay; global mapping function (GMF); no horizontal gradients
Antenna corrections	IGS20 ANTEX satellite and receiver phase center offset/variation (PCO/PCV) corrections
Other corrections	Solid Earth tides, ocean tidal loading, pole tides, phase wind-up, and relativistic effects
Positioning reference	IGS daily station coordinates

**Table 3:** Duration distribution of real-time SSR stream outage events at APM, CNE, and WHU.

Outage Duration	APM	CNE	WHU
0–1 min	173 (66.03%)	30 (25.86%)	48 (51.61%)
1–3 min	75 (28.63%)	31 (26.72%)	37 (39.78%)
3–5 min	9 (3.44%)	27 (23.28%)	5 (5.38%)
5–10 min	4 (1.53%)	27 (23.28%)	1 (1.08%)
10–15 min	0 (0.00%)	0 (0.00%)	1 (1.08%)
15–20 min	1 (0.38%)	1 (0.86%)	1 (1.08%)
>20 min	0 (0.00%)	0 (0.00%)	0 (0.00%)
Total	262 (100.00%)	116 (100.00%)	93 (100.00%)

Note: Values are event counts, with percentages of the corresponding center’s total in parentheses. Percentages may not sum exactly to 100% because of rounding.

**Table 4:** Numbers of valid satellites and samples used for real-time orbit and clock accuracy assessment.

System/ Orbit Type	No. of Satellites	Valid R-Component Samples	Valid A-Component Samples	Valid C-Component Samples	Valid Clock Samples
GPS	31	261,134	259,866	261,340	2,552,975
Galileo	25	208,086	207,134	210,792	2,015,789
BDS-3 MEO	23	190,174	189,260	190,132	1,770,331
BDS-3 IGSO	3	22,360	22,354	22,356	199,513
GLONASS	18	138,019	137,435	138,867	1,362,985

**Table 5:** Mean RMS orbit errors in the R, A, and C components for WHU real-time products.

System/Orbit Type	R RMS (cm)	A RMS (cm)	C RMS (cm)
GPS	1.73	3.39	2.76
Galileo	2.44	5.12	4.11
BDS-3 (MEO)	3.36	5.16	3.90
BDS-3 (IGSO)	13.26	10.38	11.09
GLONASS	2.68	5.89	4.24

**Table 6:** Mean clock residual STDs for WHU multi-GNSS real-time products.

System/Orbit Type	STD (ns)
BDS-3 (MEO)	0.276
BDS-3(IGSO)	0.329
GPS	0.064
Galileo	0.092
GLONASS	0.220

**Table 7:** Day-to-day variability in the accuracy of WHU real-time orbit and clock products.

System/Orbit Type	Daily R RMS (cm)	Daily A RMS (cm)	Daily C RMS (cm)	Daily Clock STD (ns)
GPS	1.74 [1.40–2.06]	3.35 [2.44–4.70]	2.74 [1.93–3.68]	0.064 [0.048–0.083]
Galileo	2.39 [1.86–3.49]	5.35 [3.08–7.34]	4.08 [2.79–5.55]	0.088 [0.071–0.161]
BDS-3 MEO	3.35 [2.98–3.94]	5.23 [3.82–6.51]	3.85 [3.03–4.86]	0.251 [0.120–0.612]
BDS-3 IGSO	13.19 [7.46–16.30]	10.57 [5.78–13.30]	10.96 [6.55–17.03]	0.280 [0.177–0.809]
GLONASS	2.62 [2.25–3.52]	5.66 [4.27–7.16]	4.05 [3.01–5.72]	0.218 [0.167–0.285]

Note: Each cell gives the median [minimum–maximum] of the 30 daily metrics.

**Table 8:** RMS 3D orbit extrapolation errors over different intervals under SSR correction extrapolation (cm).

System	0–5 min	5–10 min	0–10 min
BDS-3	1.0	2.7	2.1
GPS	3.0	7.9	5.9
Galileo	1.3	3.3	2.5
GLONASS	11.0	31.2	23.4

**Table 9:** RMS clock extrapolation errors over different intervals under SSR correction extrapolation (ns).

System	0–5 min	5–10 min	0–10 min
BDS-3	0.020	0.041	0.032
GPS	0.095	0.124	0.110
Galileo	0.031	0.061	0.049
GLONASS	0.219	0.374	0.307

**Table 10:** RMS of error-scale differences between the training model and test reference model over 0–10 min.

System	Satellite-Level RMS of Error-Scale Differences (cm)	System-Level RMS of Error-Scale Differences (cm)
GPS	0.322	0.105
Galileo	0.122	0.014
BDS-3	0.085	0.009
GLONASS	0.687	0.181

**Table 11:** RMS positioning errors and between-station distributions for the four schemes during simulated SSR outages (cm).

Positioning Scheme	0–5 min	5–10 min	0–10 min	Station-Level 3D RMS Median [Min–Max] (0–10 min)
Scheme 1	1.55/1.15/3.26/3.79	1.56/1.11/3.37/3.88	1.55/1.13/3.32/3.83	3.92 [2.41–5.55]
Scheme 2	9.00/2.59/7.63/12.08	9.77/4.06/9.68/14.34	9.39/3.40/8.71/13.26	9.78 [6.36–23.33]
Scheme 3	1.73/1.31/3.57/4.17	2.02/1.58/3.68/4.48	1.88/1.45/3.62/4.33	3.92 [2.48–7.01]
Scheme 4	1.60/1.19/3.31/3.86	1.89/1.57/3.49/4.27	1.75/1.40/3.40/4.07	3.65 [2.25–6.87]

Note: Columns 2–4 list component and 3D RMS values in the order E/N/U/3D. The final column gives the median [minimum–maximum] of station-level 3D RMS values over 0–10 min.

**Table 12:** Window-wise 3D RMS positioning errors and reduction rates for system-level and satellite-level DVI during simulated SSR outages.

DOY	Outage Start (GPST)	System-Level DVI 3D RMS (cm)	Satellite-Level DVI 3D RMS (cm)	RMS Reduction (%)
035	07:45	3.30	3.10	6.00
035	14:45	4.69	4.29	8.57
035	21:45	3.38	3.01	10.92
048	07:45	3.61	3.65	−0.85
048	14:45	8.07	7.70	4.62
048	21:45	3.93	3.53	10.14
058	07:45	3.64	3.68	−1.01
058	14:45	3.06	2.44	20.17
058	21:45	2.86	2.76	3.68

Note: Reduction rates are relative to system-level DVI. Positive values indicate improvement; negative values indicate degradation.

## Data Availability

The real-time SSR data used in this study were obtained from publicly available GNSS real-time services. The processed datasets generated during the current study are available from the corresponding author upon reasonable request. The CODE final orbit and clock products and GNSS observation data used for validation are publicly available from their respective data centers.

## Abbreviations

The following abbreviations are used in this manuscript:

ANTEX	Antenna Exchange Format
APM	Academy for Precision Measurement Science and Technology, Chinese Academy of Sciences, China
BDS	BeiDou Navigation Satellite System
BNC	BKG Ntrip Client
CODE	Center for Orbit Determination in Europe
CNE	Centre National d’Études Spatiales, France
CoM	Center of Mass
DOY	Day of Year
DVI	Direct Variance Inflation
ECEF	Earth-Centered, Earth-Fixed
GEO	Geostationary Earth Orbit
GLONASS	Globalnaya Navigazionnaya Sputnikovaya Sistema
GMF	Global Mapping Function
GNSS	Global Navigation Satellite System
GPS	Global Positioning System
GPST	GPS Time
IGS	International GNSS Service
IGSO	Inclined Geosynchronous Orbit
MAD	Median Absolute Deviation
MAE	Mean Absolute Error
MEO	Medium Earth Orbit
NTRIP	Networked Transport of RTCM via Internet Protocol
PCO	Phase Center Offset
PCV	Phase Center Variation
PPP	Precise Point Positioning
PPP-AR	PPP with Ambiguity Resolution
RAC	Radial, Along-track, and Cross-track
RMS	Root Mean Square
RTCM	Radio Technical Commission for Maritime Services
RT-PPP	Real-Time Precise Point Positioning
SISRE	Signal-In-Space Range Error
SSR	State Space Representation
STD	Standard Deviation
TDCP	Time-Differenced Carrier Phase
WHU	Wuhan University, China
